# Seeking New Information With Old Questions: Children and Adults Reuse and Recombine Concepts From Prior Questions

**DOI:** 10.1162/opmi.a.12

**Published:** 2025-07-26

**Authors:** Emily G. Liquin, Marjorie Rhodes, Todd M. Gureckis

**Affiliations:** Department of Psychology, University of New Hampshire, Durham, NH, USA; Department of Psychology, New York University, New York, NY, USA

**Keywords:** question asking, development, information search, expected information gain, learning, language of thought

## Abstract

Question asking is a key tool for learning about the world, especially in childhood. However, formulating good questions is challenging. In any given situation, many questions are possible but only few are informative. In the present work, we investigate two ways 5- to 10-year-olds and adults simplify the challenge of formulating questions: by reusing previous questions, and by recombining components of previous questions to form new questions. In Study 1, we develop a new question asking task, verify its suitability for studying question asking in children and adults, and conduct a preliminary investigation of how children and adults reuse and recombine their own prior questions. In Study 2, we experimentally manipulate exposure to another person’s questions, investigating under what conditions children and adults reuse and recombine others’ questions. Our experimental results suggest that both children and adults reuse and recombine questions, and they adaptively modulate reuse depending on how informative a question will be in a particular situation. Moreover, children reuse and recombine prior questions more frequently than adults in some cases. This work shows that prior questions provide fodder for future questions, simplifying the challenge of inquiry and enabling effective learning.

## INTRODUCTION

Starting early in development, children ask questions to gain information (Chouinard, 2007; Frazier et al., 2009; Mills & Sands, 2020; Ronfard et al., 2018). Question asking is a powerful tool for learning because questions can efficiently target information that could be difficult to obtain otherwise. For example, children ask questions to learn about causal mechanisms (Callanan & Oakes, 1992), unobservables (Fitneva et al., 2013), and generic concepts (Gelman et al., 2008). Children also use questions to learn about complex, real-world issues, like the COVID-19 pandemic (Menendez et al., 2021; Unlutabak & Velioglu, 2021) and climate change (Lee & Barnett, 2020).

Question asking is so common that it can be easy to overlook what a cognitive challenge it can be to formulate a good question. After identifying uncertainty to be resolved, a learner could ask an infinite number of questions (Coenen et al., 2019). For example, imagine a child who wants to know what is inside their wrapped birthday present. The child could ask their parent: “Is it a toy?”, “Is it something I asked for?”, “How much did it cost?”, “What is two plus two?”, and so on. These questions range from useful to irrelevant. Given the large number of possible questions, how do we find questions that efficiently help us resolve uncertainty and learn about the world? One part of this is clearly linguistic (choosing the right words to express an idea), but another is cognitive—reasoning about how to request information to resolve as much uncertainty as possible.

In the present work, we investigate how children and adults might simplify the cognitive challenge of question asking. The specific idea we explore here is that prior task-relevant experience asking questions provides fodder for future questions. For example, if a child has previously asked, “What sound does a cow make?”, they might think of this question later when encountering a novel animal (e.g., an ostrich: “What sound does an ostrich make?”). We call this **reuse**: repeating questions with the same meaning and structure, applied to new situations. Note that this could include exact repetition of the same question, even applied to the same target: for example, a child might ask, “What is that?” when pointing to a cup, then later ask, “What is that?” when pointing to a different cup. However, reuse goes beyond mere imitation, as the question is applied in a new situation to address a new uncertainty (in this example, the identity of the second cup).

In addition, even when a person decides to innovate a new question—rather than reuse a prior question—they might reuse *parts* of prior questions to form new questions, which we call **recombination**. For example, if a child previously asked, “What sound does a cow make?”, they might later ask a novel question that refers to the ostrich’s sound, like “Does an ostrich make the same sound as a robin?” In this case, a concept from the previous question (querying an animal’s sound) is recombined with other, new concepts (querying whether two things are the same) to form a novel question.

**Figure 1.**
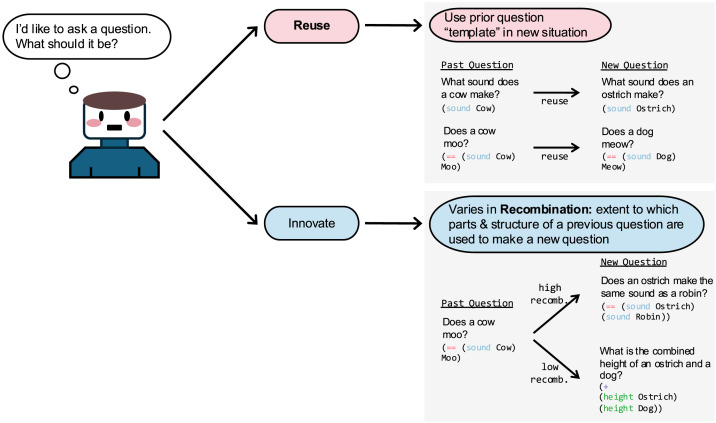
Illustration of the proposed mechanisms underlying question asking: reuse and recombination. These are operationalized using a computational framework that conceptualizes questions as computer programs. Reuse is defined as using the same question-program (i.e., function or combination of multiple functions) across situations, while optionally varying free parameters. Recombination is a continuous measure of the extent to which a question-program is similar to previous question programs (e.g., drawing on some components of a previous question, but combining them in new ways).

Drawing from memory for past questions can help constrain the search for new questions, leading to a type of cognitive economy. Thus, reuse and recombination provide a possible mechanism by which people simplify the challenge of searching the vast space of possible questions. We provide more precise definitions of these proposed mechanisms below, with examples illustrated in Figure 1.

### Question Asking Across Development

In naturalistic settings, children ask a range of sophisticated questions. For example, 4- to 5-year-olds ask domain-specific questions to learn about novel animals and artifacts (e.g., asking “What does it eat?” for an animal but “What is it for?” for an artifact; Greif et al., 2006). Preschoolers also use questions to gain causal knowledge about why things happen and how things work (e.g., “Why does it rain sometimes?”; Callanan & Oakes, 1992).

Though children ask questions to gain information from a young age, they struggle to ask maximally informative questions when provided with a particular problem to solve. Early work (e.g., Herwig, 1982; Mosher & Hornsby, 1966; Van Horn & Bartz, 1968) developed the “20-questions task” (based on the popular 20 questions game), in which children asked questions to guess a target object from an array of pictures. Rather than asking “constraint-seeking” questions, which gradually narrow down the space of possibilities by ruling out large categories of objects (e.g., “Is it a type of animal?”), many children asked “hypothesis-scanning” questions, which target a single possibility (e.g., “Is it the tiger?”). Moreover, computational models of question informativeness reveal that children’s ability to generate and identify informative questions increases between ages five and ten years (e.g., Meder et al., 2019; Nelson et al., 2014; Ruggeri & Lombrozo, 2015; Ruggeri et al., 2016, 2017, 2021, for a review see Jones et al., 2020).

Interestingly, both adults and children are better at identifying informative questions from a list of options than they are at generating informative questions themselves (Rothe et al., 2018; Ruggeri et al., 2017, but see Jirout & Klahr, 2020). This could suggest that one main bottleneck in asking good questions is the difficulty of generating possible questions in the first place.^1^

### Large-Scale Search Problems

The challenge of generating options is not unique to question asking. Many of the problems humans solve every day require searching a large space of possibilities to generate candidate options. For example, deciding what to cook for dinner, naming a pet, drawing a picture, and generating a hypothesis all require searching through a large space of possibilities (e.g., all possible dinner recipes, all possible pet names). To solve large-scale search problems, the possibilities people have considered in the past exert some influence on the possibilities they consider in the present. This influence manifests in two ways: people reuse prior possibilities, and they recombine prior possibilities to generate new possibilities. We discuss each of these in turn.

First, people reuse prior possibilities. When deciding between multiple courses of action, people are likely to think of previous actions (Bear et al., 2020; Morris et al., 2021; Phillips et al., 2019). When generating hypotheses, people prefer to maintain hypotheses they have considered in the past (Bonawitz et al., 2014). Beyond reusing one’s own ideas, people also reuse others’ ideas. For example, from a young age, children imitate others’ actions (Gergely et al., 2002; Meltzoff, 1988; Meltzoff & Williamson, 2010; Want & Harris, 2002).

Second, people innovate—but in ways that are often constrained and informed by past experience. When imagining creative products, people incorporate elements of known entities and provided examples (Smith et al., 1993; Ullman et al., 2016; Ward, 1994, 2008). When making inferences, people incorporate the output of previously-formed inferences (Dasgupta et al., 2018; Gershman & Goodman, 2014, see also Banavar & Bornstein, 2023). When generating hypotheses, people build on previously-endorsed hypotheses, rather than starting from scratch (Gelpi et al., 2020; Herbst et al., 2017; Tano et al., 2020; Zhao et al., 2024, see also Ellis et al., 2023; Tian et al., 2020). In other words, when innovating, people “recombine” their own and others’ prior ideas. Of course, innovations may vary in the extent to which they recombine prior ideas. Some innovations may be strikingly similar to prior ideas, while others may stray further. While reuse is binary (was this idea reused, or not?), recombination is continuous (to what extent does this innovated idea use concepts from prior ideas?).

How do people decide between reuse and innovation? The ideas that are most likely to come to mind are those that were high-quality and frequent in the past (Bear et al., 2020; Morris et al., 2021; Phillips et al., 2019). Using an example from Morris et al. (2021), the dinner ideas that come to mind are those that (1) one has made most frequently in the past, and (2) have been most delicious in the past (but see Ongchoco et al., 2024). However, reuse is also sensitive to the current context: from among those generated options, the dinner idea that is selected is the one that sounds best in the present situation (Morris et al., 2021). Similarly, though children and adults prefer to maintain their previous hypothesis, they switch hypotheses when the previous hypothesis no longer accounts for the observed data (Bonawitz et al., 2014). Therefore, the decision to reuse a prior idea is affected by the idea’s previous frequency and quality, as well as the idea’s quality in the current situation. To our knowledge, past research has not investigated whether recombination is also affected by these factors.

### Question Asking, Reuse, and Recombination

In the present research, we investigate whether reuse and recombination underlie question asking (see Figure 1), as they underlie other search problems. In addition, we ask whether the use of these strategies changes across development and varies according to a questions’ prior and current informational value.

Answers to these research questions will help shed light on how people solve the challenge of finding informative questions from the large space of possibilities. In particular, reuse and recombination provide a feasible alternative to searching the entire space of possible questions—rather than an exhaustive, global search, people may pursue a local search, centered around previously-used questions. Understanding (1) the developmental trajectory and (2) the context-sensitivity of reuse and recombination will help delineate the bounds of this local search process. This is especially useful in considering possible interventions to improve question asking—for example, in educational settings, where high-quality question asking is rare (Good et al., 1987; Graesser & Person, 1994). Because many proposed interventions to improve students’ question asking involve modeling high-quality questions (e.g., Birbili & Karagiorgou, 2009; King, 1990, 1991), we must understand how and when modeled questions might affect students’ later question asking. There has also been increasing interest in developing artificial intelligence systems with human-like question asking abilities (e.g., Du et al., 2017; Grand et al., 2024; Jain et al., 2017; Rao & Daumé, 2018; Wang & Lake, 2021). This endeavor requires understanding the cognitive and computational mechanisms underlying human question asking.

Preliminary evidence suggests that adults reuse and recombine previous questions. Liquin and Gureckis (2022) investigated how reuse and recombination affect adults’ questions, adapting Rothe et al.’s (2017) Battleship Task—in which participants ask questions to find hidden ships on a grid. In this prior work, participants first judged the informativeness of a set of pre-supplied questions for one set of Battleship grids, then generated their own questions for a new set of Battleship grids. After being exposed to the pre-supplied questions, adults often asked questions that either (1) had the same meaning and structure as those questions (reuse), or (2) used components of those questions (recombination), compared to a group of participants who did not receive an initial exposure to pre-supplied questions.

In addition, there is suggestive evidence for reuse and recombination in children’s question asking. Dąbrowska and Lieven (2005) analyzed 333 questions asked by two children between ages two and three; approximately 90% of these questions could be derived from minimal edits to prior speech input (including both children’s own speech and the speech of others’). For example, the question “Where can he park?” can be traced back to the child’s own prior questions of the form: “Where can [THING] park?” and “Where can he [PROCESS]?”. There is also suggestive evidence that modeling good questions can improve children’s question asking, which could be due to reuse or recombination (Birbili & Karagiorgou, 2009; Denney, 1975; Denney & Connors, 1974; King, 1990, 1991).

Despite suggestive evidence for reuse and recombination in both children and adults, the developmental trajectory of reuse and recombination is unclear. There is evidence for marked change in other aspects of question asking between childhood and adulthood (Jones et al., 2020). For example, younger children ask more hypothesis-scanning questions, which inefficiently target a single hypothesis, compared to older children and adults (Herwig, 1982; Legare et al., 2013; Mosher & Hornsby, 1966; Ruggeri & Feufel, 2015; Ruggeri & Lombrozo, 2015). In addition, children are more likely to ask redundant and unnecessary questions, which target information that has already been successfully learned from prior questions (Chai et al., 2023; Ruggeri et al., 2016). Thus, we might also expect developmental change in reuse and recombination between children and adults (and perhaps also across childhood).

For example, reuse and recombination might decrease across development, as people become more capable of sophisticated question asking. The extent to which adults reuse and recombine in other search tasks increases under time pressure (Rubino et al., 2023), suggesting that reuse and recombination are computationally efficient strategies. If these strategies are computationally efficient, we might expect children—who have limited computational resources relative to adults—to use them more often than adults. Consistent with this possibility, children’s hypothesis testing behavior can be more repetitive than adults’ (Bramley & Xu, 2023).

On the other hand, children’s questions could be less tied to previous questions than adults’. Children are more exploratory than adults (Gopnik, 2020), in seeking information (Blanco & Sloutsky, 2021; Liquin & Gopnik, 2022; Nussenbaum et al., 2023; Schulz et al., 2019), generating creative ideas (Hart et al., 2022), and considering hypotheses (Gelpi et al., 2020; Lucas et al., 2014). This includes cases of apparent reuse: adults stick to their own prior hypotheses more strongly than children do (Gelpi et al., 2020). We might expect this general exploratory tendency to extend to question formulation: children might search broadly through the space of possible questions, avoiding reuse and recombination. These possibilities motivate one of our central questions: what is the developmental trajectory of reuse and recombination?

We also consider *when* children and adults reuse and recombine questions. Prior work on other search problems (Morris et al., 2021) points to (at least) two important moderators of reuse: (1) an option’s previous quality, and (2) an option’s current quality.

If a question was particularly informative in the past, it might be more likely to come to mind again in the future—leading to higher levels of reuse for previously informative questions. Nonetheless, if a question is not informative in the *current* situation (e.g., “What sound does it make?” when learning about piece of furniture), it should not be reused—even if it was informative in the past. Indeed, people may generate questions “bottom-up”, in a way that is cued by the current state of the world (Grand et al., 2024). This mechanism would preclude reusing questions that do not apply to the current situation. Thus, we predict that reuse should be primarily sensitive to the informational demands of the present situation, but reuse might also be biased towards questions that were particularly informative in the past. Relevant to these predictions, Liquin and Gureckis (2022) found only mixed evidence that adults’ reuse was modulated according to a question’s current informativeness. They did not investigate past informativeness.

Recombination may also be affected by a question’s past and current informativeness, though this has been less well-studied in related work. If questions that were more informative in the past are more likely to come to mind in the present, it is likely that recombination will also be higher for previously informative questions. This is because a question under consideration for reuse (even if rejected in favor of a novel question) should influence the search process more than a question that does not come to mind. This would increase the level of recombination for previously informative over previously uninformative questions. We do not have specific predictions about how recombination would be affected by a question’s current informativeness.

### The Present Research

In sum, the present research addresses two questions. What is the developmental trajectory of reuse and recombination in question asking? And if children and adults do reuse and recombine questions, do they do so selectively according to a question’s previous and current informativeness?

In Study 1, we develop and test a new question asking task and computational approach. We verify that 5- to 10-year-olds and adults ask semantically diverse and informative questions in our task, and we test whether the computational approach of modeling questions as programs (see below) can capture key aspects of children’s questions. We also conduct a preliminary investigation of reuse and recombination on a trial-to-trial basis. We focused on 5- to 10-year-olds because this is a period of significant developmental change in the ability to ask informative questions (Jones et al., 2020).

Study 2 is our main investigation of reuse and recombination in question asking. In Study 2, we experimentally manipulate whether 5- to 10-year-olds and adults are exposed to a particular target question, and then test whether they later ask questions with the same meaning and structure (reuse) and ask similar, but novel, questions that draw upon components of the target question (recombination). We also manipulate the previous and current informativeness of each target question, expecting higher levels of reuse and recombination for target questions that were previously informative, and higher levels of reuse for target questions that are currently more informative.

To preview our results, we found robust evidence for reuse and recombination, with some evidence that reuse and recombination are stronger among children than adults. We found that reuse is sensitive to the current informational context in both children and adults: reuse is more common when the to-be-reused question is more informative in the present situation. However, we did not find evidence that a question’s previous informativeness affects reuse or recombination. Taken together, these results suggest that reuse and recombination provide a computationally feasible alternative to exhaustively searching the space of possible questions. Experience with one’s own and others’ questions is likely to shape question asking over time, in both children and adults.

In the following sections, we introduce our new question asking task, our computational approach, and precise definitions for operationalizing reuse and recombination. Then, we report two studies: Study 1, which develops a new question asking task and conducts a preliminary investigation of reuse and recombination, and Study 2, which uses the new task to experimentally investigate reuse and recombination.

## QUESTION ASKING TASK AND COMPUTATIONAL APPROACH

### A New Task for Studying Question Asking

**Figure 2.**
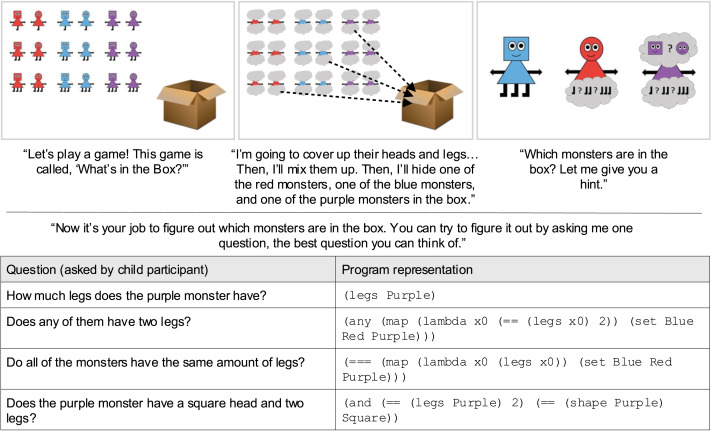
Example trial, questions, and program representations for our new question asking task.

Our question asking task (see Figure 2) builds upon established methods for studying question asking in children and adults, most notably the “Battleship task” developed by Rothe et al. (2017) and Rothe et al. (2018). In our task, participants’ goal is to identify the hidden features of a set of three monsters (one blue, one red, one purple). Monsters vary in their head shape (square or circle) and number of legs (one, two, or three). After partial information is revealed about the hidden monsters (i.e., some heads and/or legs are uncovered), participants are tasked with asking a single question that could help them figure out as much of the hidden information as possible. Questions are subject to one rule: the question could be answered with a single word, like yes, square, or two. Example questions are provided in Figure 2.

Note that this task reflects a snapshot of real-world question asking: often, a real-world question is part of a larger conversational exchange, where the learner begins with complete uncertainty, asks a question, receives an answer, then continues to ask additional questions until uncertainty is fully reduced (Frazier et al., 2009; Meder et al., 2019; Ruggeri et al., 2016). In our task, we manipulate the initial level of uncertainty on each trial, and the learner is only provided a single opportunity to ask a question. By giving each participant multiple trials with varying levels of initial uncertainty, we capture multiple “snapshots” of what question asking would look like at various points in a conversational exchange with varying levels of uncertainty. However, as a result, it is not always possible to ask a single question that is guaranteed to reveal *all* of the hidden information on a given trial. We designed the method in this way to balance methodological trade-offs: to enable a larger and more diverse sample of participants, we conducted the present studies online, with no experimenter present. As a result, we could not answer participants’ questions in real time, and thus could not simulate a full conversational exchange.

That said, this task has advantages over existing child-friendly question asking tasks (e.g., the 20-questions task) because it allows for greater semantic diversity in questions. For example, participants were not constrained to asking yes/no questions, a common feature of 20-questions tasks (e.g., Herwig, 1982; Legare et al., 2013; Meder et al., 2019; Mosher & Hornsby, 1966; Nelson et al., 2014; Ruggeri & Feufel, 2015; Ruggeri & Lombrozo, 2015; Ruggeri et al., 2019). Moreover, participants could ask questions that compared or aggregated multiple sources of uncertainty (e.g., “Is the purple monster’s head the same shape as the blue monster’s head?” or “How many monsters have two legs?”). In contrast, in traditional 20-questions tasks, there is only one source of uncertainty (a single target object).

The semantic richness of this task is especially beneficial for studying reuse and recombination, which predict semantic links between prior questions and current questions. On each trial, the participant is presented with a new uncertainty: a new set of monsters, with a unique set of features covered. This allows us to ask whether people reuse and recombine questions from previous trials—either asked by themselves (Study 1) or asked by a confederate (Study 2)—when generating questions on later trials. We define reuse and recombination formally in the next sections.

Though this task is well-suited to studying studying how people search the space of possible questions, there are some notable differences between this task and real-world question asking. First, in real-world question asking, the search space is large along two dimensions: there are many possible features to ask about (an animal’s sound, size, diet, habitat, etc.) and also many possible ways to ask about these features (by querying the feature directly, comparing the feature to other animals or objects, etc.). In our task, the latter is true—but there are only a small number of features to query (head shape and number of legs). Thus, in real-world question asking, the learner faces an even greater challenge: determining which features are worth knowing in any given situation. Our work cannot speak to how learners determine which features are worth knowing. Second, in real-world settings, one might reuse and recombine questions across highly disparate contexts—in contrast, we investigate reuse and recombination across trials of the same task. We return to this second point in the General Discussion.

### Computational Approach: Representing Questions as Programs

Because question asking is an open-ended, generative ability, it is difficult to study empirically. Prior work has therefore relied on either (1) simplified tasks where only a small number of questions are possible, but precise hypotheses can be tested using computational methods, or (2) naturalistic tasks where any question is possible, but questions must be hand-coded for descriptive analysis.

In the present research, we use a new computational approach to studying question asking—originally introduced by Rothe et al. (2017)—which gives us the best of both worlds: we can test precise hypotheses using computational methods in a task with an unbounded space of possible questions. Specifically, we represent questions as computer programs in a computational language of thought. What this means is that questions (asked by humans in natural language) are translated into computer programs (compositions of functions and parameters). We call this a question’s “program representation.” For example, intuitively, the question “What is the mean of vector x?” can be represented using the computer program mean(x). As programs, questions can be “executed” on a state of the world to determine an answer. For example, given a particular definition of x (e.g., the vector [2, 5, 3, 3]), the program mean(x) can be executed to provide an answer to the question “What is the mean of vector x?” (3.25).

This computational approach builds on the growing body of work in cognitive science in the language of thought tradition (Fodor, 1975; Piantadosi & Jacobs, 2016; Quilty-Dunn et al., 2022; Rule et al., 2020). The language of thought approach has been used to fruitfully study hypothesis and theory generation, search problems akin to question asking (e.g., Bramley & Xu, 2023; Gelpi et al., 2020; Goodman et al., 2008; Herbst et al., 2017; Lake et al., 2015; Piantadosi et al., 2012; Ullman et al., 2012; Zhao et al., 2024). Related approaches have also been used to formalize question meaning in formal semantics (Groenendijk & Stokhof, 1984, 1989; Karttunen, 1977). Therefore, this approach is promising for understanding reuse and recombination in question asking across development.

To represent questions as programs, we develop a “task-specific programming language”, with custom functions corresponding to key aspects of our question asking task. Following Rothe et al. (2017), our task-specific programming language for this task includes standard logical and arithmetic primitives (e.g., addition, and, or), custom functions for querying the head shape and number of legs of a particular monster, and methods for creating new functions. For example, the question “How many legs does the red monster have?” can be represented as (legs Red), where the first element inside a parenthetical is the function and remaining element(s) are arguments. The question “How many legs do the red and purple monsters have combined?” can be represented as (+ (legs Red) (legs Purple)). In addition, a program can define a *new* function, which can then be mapped to a number of arguments. For example, the question “Do all the monsters have a square head?” can be represented as (all (map (lambda x0 (= = (shape x0) Square)) (set Blue Red Purple))). In this example, (lambda x0 (= = (shape x0) Square)) defines a new function (where lambda indicates the definition of a new function), which takes monster x0 as an argument and returns true if the monster x0’s head is a square and false if it is not. This function is mapped to the set of all monsters (set Blue Red Purple), to determine whether each monster’s head is square. Then, the all function checks whether the resulting booleans are all true. Additional details on the task-specific programming language, including a grammar for producing questions, are provided in the Supplementary Materials.

This computational approach instantiates one possible way questions could be represented in the human mind: as structured, meaningful units, composed of a set of basic primitives. However, our goal in the present work is not to test whether the computational language of thought provides an accurate mechanistic account of human question asking (though we return to this question in the General Discussion). Instead, we use this approach as a means for operationalizing our main target of study: reuse and recombination in question asking. This is because task-specific program representations provide several key methodological advantages, especially for an open-ended task like question asking. For example, participants can ask the same question using different words: intuitively, “How many legs does the red monster have?” and “What is the red monster’s number of legs?” are the same question, in that they request information about the same feature—but they are phrased quite differently. Representing questions as programs allows us to abstract away from differences in *expression* (e.g., word choice), instead focusing on meaningful variation in questions’ *content*. Thus, in what follows, reuse and recombination are defined at the semantic level rather than the lexical and syntactic level. In addition, program representations allow for easy computation of a question’s informativeness.

### Defining Reuse and Recombination

Having introduced our computational approach, we now define reuse and recombination more precisely. **Reuse** is defined as taking a question from an earlier timepoint (applied to one uncertain situation) and using the same “question template” at a later timepoint (applied to a new uncertain situation). We define question templates by the identity and configuration of functions in a question’s program representation. Free parameters, if they exist, must have a specific “type” (e.g., color, number). We defined reuse at the level of program functions (rather than parameters) because functions map onto meaningful features in our task (head shape, number of legs).

We illustrate this with the examples from Figure 1. If a learner asks “Does a cow moo?” ((= = (sound Cow) Moo)) in one context, they may later use the same question template to ask “Does a dog bark?” or “Does a dog meow?” later. The specific animal and the specific sound are free parameters, so any animal or sound could fill those “slots.” Moreover, two identical questions share the same question template, so that repeating the exact same question across timepoints would be defined as reuse. However, “Does a dog run?” ((= = (movement Dog) Run)) would *not* count as reusing the question “Does a cow moo?” because it introduces a new function movement, and “run” is not the same type of thing as “moo” (movement vs. sound). Likewise, “Does a cow make the same sound as a robin?” ((= = (sound Cow) (sound Robin))) introduces a new function—the second instance of sound—that is not present in the original question, and thus would not constitute reuse.

For all “innovated” (non-reused) questions, we also quantified the degree to which they **recombined** elements of a prior question. How to accomplish this is less clear—most prior research investigating recombination in hypothesis generation has identified recombination by asking whether a specific component of a prior hypothesis is present or absent in a later hypothesis (Gelpi et al., 2020; Herbst et al., 2017; Zhao et al., 2024). This was also the approach used by Liquin and Gureckis (2022) in studying adults’ question recombination. Here, we aim to develop a general measure of the *degree* of recombination—not just *whether* a question uses a particular component from a previous question, but (1) how many components and (2) in how similar a format. To our knowledge, there is no standard continuous measure for recombination. However, intuitively, *similarity* may be a reasonable proxy—in general, a question that aligns more closely to a prior question’s content and structure will be more similar to that prior question, compared to a question that uses fewer shared concepts and structures.

In the present research, we measure similarity (and thus recombination) in two ways, one determined a priori and grounded in our computational approach, and one determined in light of our empirical results. The first, “grammar-based similarity” is measured through tree edit distance, which captures the number of edits needed to get from one question’s program representation to another question’s program representation. We provide further information on this measure in the Supplementary Materials. Briefly, we represent each question program as a tree: functions and arguments are nodes, and arguments are the children of their parent functions. We then compute the minimum number of edits (insertions, deletions, or relabelings) needed to go from one tree structure to another, using the Zhang-Shasha algorithm (Zhang & Shasha, 1989). Changing one argument to another argument of the same type does not count as an edit. Using the examples from Figure 1, the question “Does an ostrich make the same sound as a robin?” ((= = (sound Ostrich) (sound Robin))) is two edits away from the question “Does a cow moo?” ((= = (sound Cow) Moo)). This is because the argument Moo must be relabeled with the function sound, then the new argument Robin must be inserted. The argument Cow is replaced with Ostrich, but this does not count as an edit since these are both arguments of the same type (animals). Tree edit distance is a reverse-scored measure of similarity: questions that are more distant from each other are less similar. Therefore, we norm this measure to range from 0 to 1, with 0 indicating the highest values of tree edit distance present in the data (minimal similarity) and 1 indicating a tree edit distance of zero (maximal similarity).

Using tree edit distance as a measure of grammar-based similarity implies several assumptions about how recombination works. Specifically, it implies that questions are represented in a language of thought, and recombined by (1) starting from a previously-used question, then (2) making incremental edits to it. However, other representations and algorithms could underlie recombination—for example, one might cache previous questions as new primitives in the grammar (see Cheyette & Piantadosi, 2017; Zhao et al., 2024). In addition, recombination could result from a algorithm that is *not* grounded in a grammar-like representation. For example, large language models—which have no underlying grammar—might reuse similar outputs across timepoints simply due to learned statistical regularities.

In fact, we noticed after conducting Studies 1–2 that grammar-based similarity failed to capture certain aspects of question similarity. For example, “Does the purple monster have two legs?” ((= = (legs Purple) 2)) and “Do any of the monsters have two legs?” ((any (map (lambda x0 (= = (legs x0) 2)) (set Blue Red Purple)))) are intuitively quite similar, but have a large tree edit distance. Generally, tree edit distance can produce low similarities when one question’s program representation is significantly longer than the other. This led us to wonder whether one or more of the assumptions underlying grammar-based similarity were invalid.

We conducted an additional study (reported in the Supplementary Materials) to determine whether grammar-based similarity—along with four additional similarity measures—captured adults’ notions of question similarity. We reasoned that the cognitive process involved in forming a similarity judgment might be the same cognitive process involved in recombination, as similarity judgments track both surface-level and relational similarity (Gentner & Markman, 1997; Goldstone et al., 1991). We found that adults’ similarity judgments were far better captured by a black-box machine learning model of similarity, which estimated the cosine similarity between pretrained sentence embeddings for the semantic meaning of each question (using the question grammar to preprocess questions, eliminating differences in expression).^2^ We refer to this as “text-based similarity.” Text-based similarity was strongly and significantly correlated with adult similarity judgments, *r*(98) = 0.88, *p* < .001, while grammar-based similarity was not significantly correlated with adult similarity judgments, *r*(98) = 0.11, *p* = .27.

Note that the superiority of text-based similarity in capturing adult similarity judgments does not imply that human question asking is *not* well-described by a computational grammar. Human minds could represent, generate, and recombine questions using a mental grammar—but using different algorithms from those assumed by our grammar-based similarity measure. Text-based similarity allows for a more expansive notion of “recombination,” where any semantic similarity between two questions counts as recombination.

That said, our main goal in the present research is not to test the language-of-thought grammar as a mechanistic account of human question asking, but rather to use it as a methodological tool for operationalizing reuse and recombination. Thus, in the studies that follow, we report results using both measures of similarity: grammar-based similarity and text-based similarity. The former has the advantage of being interpretable in terms of our question grammar. It is more closely aligned with our definition of recombination (the extent to which a question uses one or more parts of a previous question in a similar format) and it is the measure we preregistered in Study 2. However, it makes stronger assumptions about the process of question generation. The latter has the advantage of more closely resembling adult similarity judgments and capturing a broader notion of recombination, but we selected it post hoc. We return to a comparison of these measures in the General Discussion.

## STUDY 1

The primary goal of Study 1 was to develop and test a novel task for investigating question asking in 5- to 10-year-olds and adults. We use the question asking task described above. Our focus was on verifying that (1) the majority of children’s and adults’ questions could be formalized in a task-specific programming language, which we use to operationalize reuse and recombination, and (2) both children and adults asked questions that vary in semantic content and informativeness.

We also conducted exploratory analyses to investigate sequential dependence in question asking from earlier trials to later trials. This is likely to reflect one manifestation of question reuse and recombination: when asking a question, do people draw upon their own previous questions? And how does this change across development?

Further methodological details, data, and analysis scripts can be found at https://osf.io/r3usj/ and https://github.com/emilyliquin/question-reuse.

### Methods

#### Participants.

The participants in Study 1 were 98 children (29 5- to 6-year-olds, 36 7- to 8-year-olds, and 33 9- to 10-year-olds; 53 girls and 45 boys) recruited and tested online through the PANDA platform for remote research (discoveriesinaction.org; Rhodes et al., 2020). Participants also included 36 adults (ages 20 to 60; 20 men, 14 women, 1 non-binary/genderqueer individual, 1 unspecified) recruited and tested online through Prolific. All adult participants and parents of child participants provided informed consent, and all child participants assented to participate by clicking a response option on the screen. Child participants had the following racial/ethnic backgrounds: 65% White, 11% multiracial, 9% Black, 7% Asian, 2% other, and 6% Hispanic of any race (1% not specified). Adult participants had the following racial/ethnic backgrounds: 70% White, 17% Black, 3% Asian, 3% multiracial, 11% Hispanic of any race.

We recruited and tested an additional 7 child participants, who were excluded for data saving issues (*n* = 3), repeating the study more than once (*n* = 1), and missing audio recording (*n* = 3). We recruited and tested an additional 4 adult participants, who were excluded for data saving issues (*n* = 1), no recorded Prolific ID (*n* = 1), failure to confirm working audio (*n* = 1), and failure to complete a simple attention check task (sorting pictures of apples and bananas into named bins; *n* = 1).

#### Materials.

In the task, participants asked questions to determine the features of hidden “monsters”. We created 18 cartoon monsters that varied along three features: color (red, blue, or purple), head shape (circle or square) and number of legs (one, two, or three). Each trial in the main question asking task involved presenting a set of three monsters—one of each color. Some or all of the monsters in the set had features obscured by clouds. For example, a cloud might cover the head of the red monster, making it unclear what shape it was. We constructed 14 possible trial arrays, each of which had a unique number of heads and legs covered by clouds, and thus differing levels of uncertainty about the set of monsters. The 14 trial arrays represented each way of covering between 0 and 3 monsters’ heads and between 0 and 3 monsters’ legs, excluding situations where no features were covered and where all features were covered. Within each level of uncertainty (i.e., number of heads and legs covered), we constructed the trial array by first randomly selecting the three monsters’ features, then randomly selecting which specific monsters’ heads and/or legs would be covered, then re-arranging the monsters from fewest features hidden (farthest left) to most features hidden (farthest right).

Figure 2 provides an example trial where two legs are hidden (the red monster’s and the purple monster’s) and one head is hidden (the purple monster’s). Each participant viewed four of these 14 possible trial arrays, presented in a random order, and were prompted to ask a single question to help reveal the hidden features on each trial.

#### Procedure.

The study was self-guided, without moderation by an experimenter. Children spoke their responses aloud, and video/audio of the child participating was recorded by the experiment interface. Adults provided responses by typing on a keyboard into a text box. All instructions and trial information were provided via pre-recorded audio narrations accompanied by images and animated videos.

At the start of the experiment, child participants were familiarized with providing verbal responses: they were asked two questions (their age and favorite color), which they were prompted to answer when a picture of a microphone appeared on the screen.

Next, participants were introduced to the full set of 18 monsters. This introduction also highlighted the three feature dimensions (e.g., “some of the monsters have square heads, and some of the monsters have circle heads”). Participants then completed eight trials where they were prompted to click on a monster with a particular feature (e.g., “Can you click on the monster with a square head?”). This served to familiarize participants to the relevant features.

Next, participants were introduced to the question asking task, in which they would ask a question to identify which monsters were hidden in a box. Participants were asked to follow one rule: the answer to each question had to be one word (for example, “yes,” “square,” “two,” or “purple.”) This rule prevented questions like, “What do all of the monsters look like?” To facilitate their understanding of the task, participants were provided with four example questions. There were no hidden monsters on the screen when the example questions were provided.

Finally, participants completed four trials of the question asking task, where each trial contained a unique trial array selected at random from the 14 trial arrays described above. On each trial, participants watched an animation in which all 18 monsters’ features were occluded, the monsters were shuffled, one monster of each color was selected, and then the three selected monsters were hidden in a box (see Figure 2). Then, participants were provided a “hint”: some features were revealed while others’ remained occluded, corresponding to one of the 14 trial arrays described above. After seeing the trial array, participants’ task was to ask a single question that could help them figure out which monsters were in the box, the best question they could think of.

After each question, the hidden features were revealed, indirectly answering the participant’s question. Next, participants watched an animal video to maintain engagement. Then, participants proceeded to the following trial, until all four trials were completed.

At the end of the study, adult participants and parents of child participants filled out a brief demographic form.

#### Data Preprocessing.

Children’s responses were transcribed from video by a trained research assistant, with all trial information (i.e., which trial array each response was directed towards) masked. Then, the first author (1) coded each response as valid, invalid (e.g., off-task, non-question, does not follow rule to have a one-word answer), or ambiguous (multiple possible interpretations), and (2) translated (by hand) each valid question into the task-specific programming language described below.

Among children, 29 trials were skipped or had poor audio quality of the 392 total trials across all 98 participants. Among adults, 1 trial was skipped of the 144 total trials across all 36 participants. We removed these trials from all further analysis. Of the 363 transcribed responses produced by all children (on 363 total trials), 17% were invalid responses and 9% were ambiguous questions. Of the 143 responses provided by all adults (on 143 total trials), 4% were invalid responses and 8% were ambiguous questions. Finally, of adults’ remaining 126 questions, nine could not be translated into the prespecified task-specific language while maintaining their intended meaning. Excluding these questions (e.g., “What monster, if any, is the only one with their unique head shape?”) left a final sample of 271 questions from 87 children (asked across 271 total trials) and 117 questions from 36 adults (asked across 117 total trials). See Supplementary Materials for additional examples of excluded responses.

Because the study was conducted remotely and without an experimenter present, we coded whether anyone interfered with a child’s response during the four question asking trials, for roughly 20% of the children who contributed to the analyzed dataset (18 children). Interference rate was reasonably low (5.6% of trials), and most identified cases of interference likely had minimal influence on the child’s response (e.g., a parent reminding the child to ask only one question or to direct their question to the camera rather than the parent). Inter-rater reliability for a subset of 10 videos (40 trials) was substantial (agreement = 95%; Cohen’s kappa = 0.64).

#### Task-Specific Language.

Children’s and adults’ questions were modeled as programs in a task-specific programming language adapted from Rothe et al. (2017), as described above. See Figure 2 for examples, and the Supplementary Materials for a full specification of the task-specific language. The first author translated each question by hand into the task-specific language. Notably, many questions could be represented several different ways in the task-specific language. For all questions, we ensured that translations were consistent across multiple instances of the same question.

To validate the human-translated program representations, we developed a prompt for GPT-3.5-turbo (Brown et al., 2020) to back translate each human-translated program into an English natural language question. Recent research has used large language models (LLMs) to translate from natural language to program representations (Wong et al., 2023), including in question asking tasks (Grand et al., 2024). Given these successes, we expected that an LLM could also successfully back translate from a program representation to natural language. Notably, however, this validation procedure did not presuppose that the LLM would suceed in 100% of cases (and in fact, it did not, see Supplementary Materials). Instead, this procedure was developed to flag translations that required further human inspection. A trained research assistant compared each of the LLM’s back translations to the corresponding original question. In cases where original and back translated questions did not match, the first author then checked the original translations for errors (and updated them as needed). In nearly all cases of mismatch, this was the result of LLM (rather than human) error. Thus, this procedure validated that the human translations were nearly error-free—and allowed us to correct the few errors that were detected prior to data analysis. See Supplementary Materials for further details.

#### Modeling Question Informativeness.

One of our goals for Study 1 was to investigate whether children and adults can ask reasonably informative questions in our novel task (possibly with improvement across development, mirroring prior work). Answering this question requires a precise measure of question informativeness. Following prior work on children’s and adults’ question asking (e.g., Liquin & Gureckis, 2022; Rothe et al., 2018; Ruggeri et al., 2016), we formalize question informativeness using *Expected Information Gain* (EIG; Lindley, 1956), which defines informativeness as the expected reduction in uncertainty (i.e., entropy) from receiving the answer to a question.^3^ In our task, questions are informative to the extent that they are likely to narrow down the hypothesis space of possible hidden monsters. See Supplementary Materials for the formal definition of this measure in the context of our task.

#### Quantifying Reuse and Recombination.

Study 1 also investigated reuse and recombination, from earlier trials to later trials. We quantified reuse and recombination as described previously. Reuse and recombination were quantified programmatically, using custom R and Python scripts (which can be found at https://github.com/emilyliquin/question-reuse).

### Results

#### Statistical Approach.

Unless otherwise noted, the statistical analyses below use mixed-effects regression models, with random intercepts to account for the nested structure of the data (questions within participants, and questions within trial arrays). Categorical predictors (e.g., age group) were dummy coded. We estimate the statistical significance of fixed effects using likelihood ratio tests, comparing a model including the fixed effect of interest to a model excluding it. In addition, we report unstandardized regression coefficients with 95% confidence intervals. For logistic regression models, we transform the regression coefficients to produce odds ratios (OR).

For all models involving age, we followed a two-step procedure. First, we fit a model testing the effect of mean-centered age in months, using only the child data. If effects of age within childhood (main effects or interactions) were non-significant, we subsequently fit a model comparing children (collapsing across age) and adults. However, if any effects of age within childhood were significant, we subsequently fit a model comparing 5- to 6-year-olds, 7- to 8-year-olds, 9- to 10-year-olds, and adults. We chose these age bins to have similar numbers of participants in each bin. Below, we report only the final regression model from this multi-step analysis. Thus, for some analyses, we report results from children as a group, while for other analyses, we report results from three separate child age bins.

#### Evaluation of Question Asking Task.

**Figure 3.**
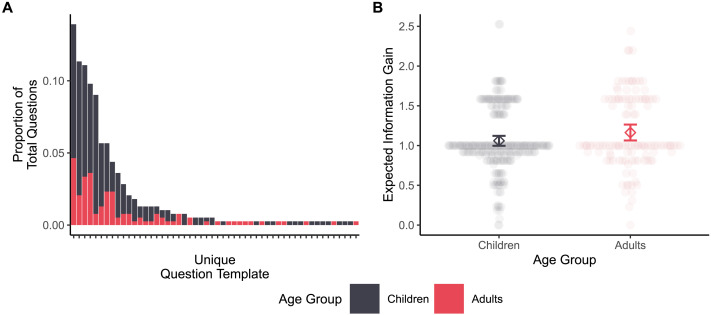
**A:** Children and adults asked a variety of questions. Each bar corresponds to a different question template (defined by a unique program representation). Many questions were asked by both children and adults, while some (less frequent) questions were unique to one age group. **B:** Children and adults asked similarly informative questions. Diamonds indicate mean expected information gain with bootstrap 95% confidence intervals, and transparent points indicate the expected information gain of individual questions asked by individual participants.

First, we evaluated whether participants asked semantically diverse and informative questions in our novel question asking task. We found that both children and adults asked a variety of questions. For each of the 14 trial arrays, we counted the number of unique question templates (e.g., “Does the [color] monster have [number] legs?”) used by children and adults. In total, children used 38 unique question templates (across 271 total trials), while adults used 34 unique question templates (across 117 total trials). Examples of children’s questions for one of the 14 trial arrays are provided in Figure 2. On average, 45% of children’s questions on each trial used a unique template, compared to 67% of adults’ questions. A *t*-test found a significant difference in the proportion of unique question templates between children and adults, *t*(26) = −3.14, *p* = .004, Δ*M* = −0.22, 95% CI [−0.37, −0.08]. Thus, a variety of question templates were used, even within a single trial—though adults’ questions were slightly more variable than children’s. That said, the most commonly-used question templates were asked by both children and adults (see Figure 3A).

We also found that adults asked slightly more complex questions than children. In particular, we investigated (1) the number of unique functions contained in each question’s program representation, and (2) the number of total functions contained in each question’s program representation (in both cases excluding lambda as a function, as it always co-occurred with map). For example, the question “Do all the monsters have a different number of legs and the same head shape?” was quite complex according to both measures, while the question “What shape is the red monster’s head?” was quite simple according to both measures. There was a significant effect of age group (5- to 6-year-olds, 7- to 8-year-olds, 9- to 10-year-olds, adults) on the number of unique functions in a question, *χ*^2^(3) = 16.52, *p* < .001, with question complexity increasing across development (see Supplementary Materials for regression coefficients). Adults’ questions also used slightly more total functions than children’s questions, though the effect of age group (children, adults) on total functions was not significant, *χ*^2^(1) = 3.55, *p* = .06, *b* = 0.68, 95% CI [−0.03, 1.39].

Next, we found that both children and adults asked reasonably informative questions. On average, children’s questions had EIG 1.06 (*SD* = 0.39), while adults’ questions had EIG = 1.16 (*SD* = 0.46), see Figure 3B. A mixed-effects linear regression revealed no evidence for a difference in question informativeness as a function of age group (children, adults), *χ*^2^(1) = 3.75, *p* = .053, *b* = 0.12, 95% CI [−0.001, 0.24]—adults’ questions were slightly, but not significantly, more informative than children’s. These results are striking in light of the robust shift in question informativeness across development reported in prior work (for a review, see Jones et al., 2020), and we discuss this further in the General Discussion. Nonetheless, this suggests that children and adults alike asked reasonably informative questions in this question asking task.

That said, questions were not as informative as they could have been. For each question asking trial, we estimated the EIG of the most informative possible question (formally, one that would fully reduce entropy to zero, see Supplementary Material).^4^ If all participants in our dataset had asked a perfectly informative question on each trial, the expected average EIG would be 3.71 (compared to the observed average 1.09). Even the most informative question asked in each trial by any of our participants fell far short of the maximum possible EIG, averaging 1.82 across the 14 different trial arrays.

Inspired by Rothe et al. (2018), we also investigated the context-sensitivity of participants’ questions. An informative question in one context could be uninformative in another context. Do children and adults tailor their questions to the context, or do they ask general-purpose questions that are informative across contexts? We permuted the trial type associated with each question in 1000 simulated datasets. The average EIG of questions in the original dataset was higher than the average EIG in all 1000 permutation datasets, for both children’s and adults’ questions (*p*s < .001). This suggests that children’s and adults’ questions were not general-purpose, but rather were context-sensitive.

#### Reuse and Recombination Across Trials.

After collecting the Study 1 data, we realized that these data might bear on our main research questions regarding question reuse and recombination. In particular, reuse and recombination might unfold across the four trials, such that the questions a participant asked on later trials could be shaped by the questions they asked on earlier trials.

To explore this possibility, we investigated reuse and recombination across trials, including any trials where a participant asked a valid question on at least one earlier trial (as well as the present trial). In other words, the following analyses excluded (1) the first valid question asked by each participant, or (2) any valid question that was not preceded by at least one other valid question. This left 265 trials (184 for children, 81 for adults). For all analyses in this section, mixed-effects models included by-participant random intercepts, with no other random effects.

**Figure 4.**
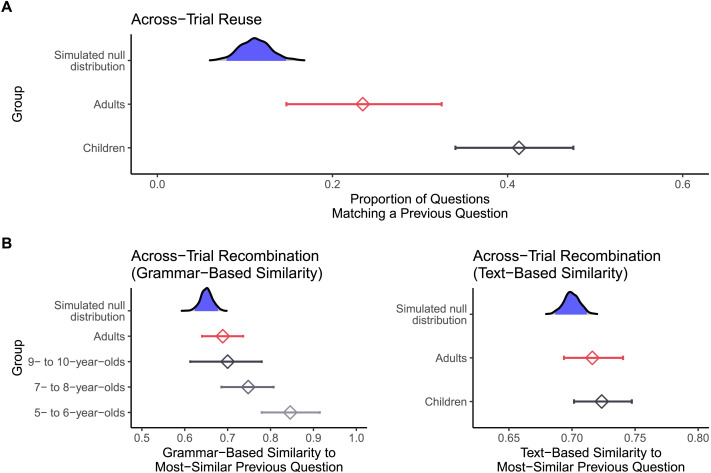
Reuse (**A**) and recombination (**B**) from earlier trials to later trials in Study 1. For all figures, density plots show the simulated null distribution, with randomly sampled “previous trials” matched to the trial structure of the true data. The middle 95% of the null distribution is shaded in blue. Diamonds with error bars represent the mean and bootstrap 95% confidence interval of observed reuse/recombination for each age group. *X*-axes truncated for ease of visualization.

Both children and adults reused questions across trials, but there was evidence that children reused their own prior questions more than adults. Children repeated an earlier question template on 41% of trials where such repetition was possible, while adults repeated an earlier question template on 24% of such trials. The effect of age group (children, adults) on the probability of reusing one’s own previous question template was significant, *χ*^2^(1) = 8.12, *p* = .004, *OR* = 0.44, 95% CI [0.24, 0.79], with adults having a lower predicted odds of reusing earlier question templates than children (see Figure 4A).

One deflationary account is that these apparent cases of reuse merely reflect the structure of our task: if the same question templates just happen to be informative across multiple trials, then repeating a question template may not be evidence for reuse. Instead, it may simply indicate that people generate informative questions on each trial (independent of all previous trials). To rule this out, we compared the observed rate of question reuse to a simulated null distribution. For each valid trial in the above analysis, we replaced each of the previously-asked questions with a randomly selected question, drawn from all questions asked for that same trial array (by any participant). We repeated this procedure for 1000 simulated datasets, and extracted the proportion of reuse from each. This procedure results in a null distribution that represents the amount of reuse that would be expected if question asking on each trial is independent of all previous trials, but still respects each trial’s informational demands. If reuse simply reflects the task’s structure, we would expect the observed rate of reuse to fall within the null distribution. In contrast, providing evidence for reuse, the observed rates of reuse (for both children and adults) were more extreme than all 1000 simulated estimates (one-tailed *p*s < .001; see Figure 4A).

We also found evidence for recombination, with some evidence that children’s questions were more similar to their own prior questions than adults’ (see Figure 4B). We computed the similarity between each question and all previous questions asked by the same participant—excluding trials that were classified as instances of reuse (as our definition of recombination only applies to non-reused, innovated questions)—then took the maximum similarity value. Therefore, we analyzed the degree of recombination between each asked question and the *most similar* previously-asked question. As measured by grammar-based similarity, the degree of recombination decreased across development: the effect of binned age group (5- to 6-year-olds, 7- to 8-year-olds, 9- to 10-year-olds, adults) on grammar-based similarity was significant, *χ*^2^(3) = 9.67, *p* = .02, with similarity to one’s own prior questions decreasing across development (e.g., adults vs. 5- to 6-year-olds: *b* = −0.16, 95% CI [−0.26, −0.05], see Supplementary Materials for full regression results). However, as measured by text-based similarity, there was no evidence for differential levels of recombination in adults compared to children, *χ*^2^(1) = 0.15, *p* = .70, *b* = −0.01, 95% CI [−0.05, 0.03].

Again, we ruled out that recombination merely reflected the trial structure of our task. As above, we constructed a simulated null distribution by repeatedly replacing previously-asked questions with randomly sampled trial-array-matched questions, with the additional stipulation that sampled previous-trial questions could not match the current-trial question template (thus excluding instances of reuse). For grammar-based similarity, the degree of recombination in all age groups (5- to 6-year-olds, 7- to 8-year-olds, 9- to 10-year-olds, adults) was more extreme than the large majority of simulated estimates (one-tailed *p*s < .01; see Figure 4B). This was also true for text-based similarity (one-tailed *p*s < .01; see Figure 4B). Thus, both children and adults recombined their own prior questions, according to both measures of recombination.

### Discussion

Study 1 was designed to evaluate our novel question asking task. Consistent with our goals for the task, the great majority of participants’ questions could be represented as programs in the task-specific language. Both children and adults asked a variety of questions, though adults asked slightly more complex and variable questions than children did. In addition, both children’s and adults’ questions were reasonably informative and highly context-specific. Together, this suggests that this task is suitable for studying question asking across development, and it elicits a sizable variety of natural language questions while enabling precise quantitative analyses.

In addition to validating our task, we also investigated reuse and recombination on a short timescale: from earlier trials to later trials. We found evidence for reuse and recombination in both children and adults. Critically, reuse and recombination could not be explained by the trial structure of the task. Simulation analyses suggested that we would observe much lower rates of reuse and lower amounts of recombination if participants had simply asked a reasonable question on each trial. This suggests that participants’ own prior questions may have influenced the questions they generated later.

We also found some evidence that children were more likely to reuse and recombine prior questions compared to adults. Specifically, children reused their own prior questions on 41% of trials, compared to 24% of trials in adults. In addition, as measured by grammar-based similarity (but not text-based similarity), children’s innovated questions were more similar to their own prior questions. This suggests that children may be especially likely to reuse (and perhaps recombine) prior questions to simplify the challenge of finding informative questions.

These results are suggestive, but all analyses on reuse and recombination were exploratory. Moreover, reuse and recombination across trials could be explained by participants’ idiosyncratic preferences for particular question templates, rather than a specific effect of prior experience. Thus, in Study 2, we conduct a preregistered experimental test of reuse and recombination across development. In addition, we investigate *when* people reuse and recombine questions, focusing on questions’ prior and current informativeness.

## STUDY 2

Study 2 addresses our central research questions: How do reuse and recombination compare across development? And when do children and adults reuse and recombine questions? To answer these questions, we manipulate whether or not participants were exposed to a particular target question, then we ask how that exposure (or lack thereof) affects later question asking. This manipulation is akin to prior efforts to improve children’s question asking ability by “modeling” good questions (e.g., Birbili & Karagiorgou, 2009; King, 1990, 1991). However, in the present research, beyond investigating the effect of modeling on later question informativeness, we investigated the effect of modeling on the *semantics* of later questions—specifically, how children and adults reuse and recombine the modeled question.

To do this, we manipulated whether or not participants saw a single “practice trial” before completing the question asking task: participants in the exposure condition saw the practice trial, while those in the no-exposure condition did not. In the practice trial, a confederate asked a question to resolve uncertainty about the hidden monsters. We randomly assigned each participant in the exposure condition to hear the confederate ask one of two possible “target questions” in the practice trial, both of which were relatively rare in Study 1: “How many monsters have a square head?” or “How many legs do all the monsters have combined together?”

Though participants in the no-exposure condition did not complete the practice trial and thus were not exposed to any particular question, the trials they saw in the subsequent question asking task were matched to a particular target question condition. Thus, the “target question” for each participant in the no-exposure condition was defined as the question that they would have been exposed to, had they been the exposure condition. This provides an ideal control condition: if participants ask the target question and/or semantically related questions in the question asking trials for any reason having to do with the structure of the trials themselves (e.g., because the target question is informative), then we would expect participants in *both* conditions to ask these questions at similar rates. However, if participants leverage the question from the practice trial to generate future questions, then participants in the exposure condition should ask the target question (reuse) and/or similar questions (recombination) more often than participants in the no-exposure condition. Thus, to establish reuse and recombination, we focus on comparisons between the no-exposure and exposure conditions. An effect of exposure condition would demonstrate that a single, minimal exposure to another person’s question influences people’s later question asking. To investigate age differences in reuse and recombination, we test the interaction between exposure condition and age group, which indicates whether the size of the exposure effect differs across ages.

We also manipulated how informative the target question was both (1) in the practice trial (“prior informativeness”) and (2) during later question asking (“current informativeness”). We do so by varying the trial array (i.e., which monsters’ features are hidden) in both the practice trial and the later question asking trials. In some cases, the target question would be very likely to reveal much of the hidden information, while in other cases, it would not. We predicted that both prior and current informativeness may influence reuse and recombination. In particular, people might be more likely to reuse and recombine questions that were more informative in the past, as these may be more likely to come to mind in the present (Bear et al., 2020; Morris et al., 2021; Phillips et al., 2019). However, participants might also selectively choose to reuse questions only in situations where they are currently informative (Morris et al., 2021).

Research questions and analyses for Study 2 were preregistered (https://osf.io/wjrvt/). All deviations from our preregistration are noted below. Further methodological details (e.g., task instructions), data, and analysis scripts can be found at https://osf.io/r3usj/ and https://github.com/emilyliquin/question-reuse.

### Methods

#### Participants.

Participants were 219 children recruited and tested online through a the PANDA platform for remote, unmoderated research (discoveriesinaction.org; 66 5- to 6-year-olds, 78 7- to 8-year-olds, and 75 9- to 10-year-olds; 112 girls and 107 boys) and 179 adults recruited and tested online through Prolific (ages 19 to 67; 101 men, 76 women, 2 non-binary/genderqueer). All adult participants and parents of child participants provided informed consent, and all child participants assented to participate by clicking a response option on the screen. Child participants were 58% White, 17% Asian, 11% multiracial, 5% Black, and 11% Hispanic of any race (2% not specified). Adult participants were 77% White, 9% Black, 4% Asian, 2% multiracial, 1% American Indian/Alaska Native, 8% Hispanic of any race (1% not specified).

Participants were randomly assigned to one of two exposure conditions (no-exposure, exposure), one of two previous quality conditions (previously-informative, previously-uninformative), and one of two target question conditions (“How many monsters have a square head?” or “How many legs do all the monsters have combined together?”). We assigned conditions so that the exposure condition (which was our main condition of interest) had about twice as many participants (*n* = 271) as the no-exposure condition (*n* = 127). Within each exposure condition, an approximately equal number of participants was assigned to each combination of previous quality condition and target question condition. As explained below, though participants in the no-exposure condition were assigned to a previous quality condition and target question condition, they were not actually exposed to any target question. These condition assignments were used to determine the trial arrays participants saw during the instructions and question asking task—which were matched to the trial arrays seen by participants in the corresponding exposure condition.

We excluded an additional 12 children for repeating the study more than once (*n* = 5), having already participated in Study 1 (*n* = 1), or missing audio or video recording (*n* = 6). We excluded an additional 29 adults for for data saving issues (*n* = 2), no recorded Prolific ID (*n* = 14), failure to confirm working audio (*n* = 1), and failure to complete a simple attention check task (*n* = 12).

#### Procedure.

**Figure 5.**
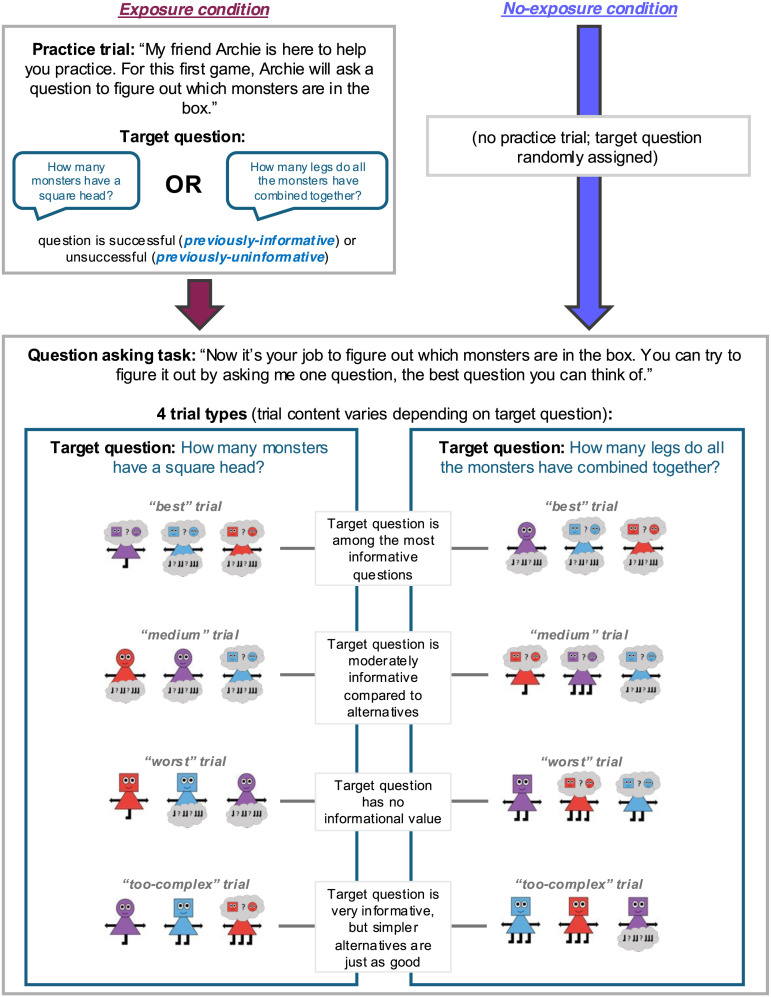
Overview of Study 2 procedure.

See Figure 5 for an overview of the procedure. As in Study 1, participants were first introduced to the monsters (including their various features) and the question asking task. All participants saw one illustrative trial array in the instructions phase, which was distinct from all trial arrays in the later question asking task.

Next, participants in the **exposure condition** (but not the **no-exposure condition**) completed a “practice trial” with the illustrative trial array from the instructions phase. In this practice trial, a confederate (a prerecorded video of an adult research assistant) asked a question (the **target question**: “How many monsters have a square head?” or “How many legs do all the monsters have combined together?”, depending on target question condition). Participants were prompted to answer the question by choosing from several possible answers. If participants clicked an incorrect answer, they were corrected.^5^ Finally, the confederate guessed the hidden monsters, taking into account the provided answer.

We manipulated the informativeness of the target question in the practice trial by varying the trial array in the practice trial between-subjects. Therefore, for each target question, some participants viewed evidence that it was informative and others viewed evidence that it was uninformative. In the **previously-informative condition**, the trial array was selected so that the target question was highly informative. In addition, after the participant answered the confederate’s question, the confederate made a correct guess about the hidden monsters. In contrast, in the **previously-uninformative condition**, the trial array was selected so that the target question was less informative. In addition, after the participant answered the confederate’s question, the confederate made an incorrect guess about the hidden monsters. We manipulated both informativeness and whether the confederate’s guess was correct because prior research suggests that children sometimes attend to question asking success (i.e., whether the hidden information was correctly identified) over question informativeness (Török et al., 2023).

Finally, participants (in all conditions) completed four question asking trials. The trial arrays used for these four trials varied depending on the assigned target question condition, so that the target question had varying levels of informativeness across the four trial arrays. Drawing from all questions asked in Study 1, the target question was among the most informative questions for one trial array **(“best” trial)**, a moderately informative question for one trial array **(“medium” trial)**, and an uninformative question for one trial array **(“worst” trial)**. For another trial array, the question was maximally informative, but more complex than needed **(“too-complex” trial)**. We refer to these as “trial types.” The order of the four trial types was randomized for each participant.

#### Data Preprocessing.

Data preprocessing was identical to Study 1. As preregistered, we excluded all trials that were skipped or had poor audio quality (2% of trials), responses that were non-questions, did not follow the rules, or were off-task (11% of trials), ambiguous questions (6% of trials), and questions that could not be translated into the task-specific language (<1% of trials). This left a sample of 642 questions (asked on 642 trials) from 196 children and 633 questions (asked on 633 trials) from 176 adults, which exceeded our preregistered target of 600 questions per age group. We provide more information about exclusions in the Supplementary Materials.

Like Study 1, we coded whether anyone interfered with the child’s response during the four question asking trials for roughly 20% of the children who contributed to the analyzed dataset (40 children). Interference rate was again low (6.2% of trials) and qualitatively mild (e.g., repeating the instructions; approving of child’s whispered question and encouraging them to ask it aloud). Inter-rater reliability for a subset of 20 videos was substantial (agreement = 96%; Cohen’s kappa = 0.75).

#### Quantifying Reuse and Recombination.

In this study, our main analyses concern the extent to which participants reused and recombined the *target question* (though we also analyze the extent to which participants reused and recombined their own previous questions, replicating Study 1).

For reuse, in the “How many monsters have a square head?” target question condition, both this exact question and “How many monsters have a circle head?” used the same question template as the target question. In the “How many legs do all the monsters have combined together?” target question condition, only this exact question matched the target question (because the question has no free parameters). In the analyses below, we refer to these as “target-matching questions.” This is because participants in the no-exposure condition were never exposed to the target question, and thus using the target question did not indicate “reuse” in this condition. Instead, reuse is measured by whether a higher proportion of target-matching questions are asked in the exposure condition relative to the no-exposure condition. We call this the “reuse effect.”

Like Study 1, recombination was measured using both grammar-based similarity and text-based similarity. In this case, we computed the similarity between the target question and each participant’s question on each trial. In the analyses below, we refer to this as “target-question similarity.” This is because participants in the no-exposure condition were never exposed to the target question, and thus have nothing to “recombine.” Instead, recombination is measured by whether participants in the exposure condition ask questions with higher target-question similarity, relative to participants in the no-exposure condition. We call this the “recombination effect.”

### Results

#### Statistical Approach.

All analyses use mixed-effects regression. We deviated from our preregistered analysis plan to better align with best practices for mixed-effects regression analyses (though the preregistered analyses produce highly similar results). We initially preregistered a separate regression model to test each of our predictions, without controlling for other manipulated variables. Instead, we report below the minimal set of models needed to test all predictions, allowing us to control for all manipulated variables in each model. In addition, we initially preregistered that models would include maximal random effects structure, accounting for clustering by participant, target question, and trial type. In practice, the maximal models rarely converged, nor did most of the simpler models that retained random slopes. Moreover, since target question and trial type were a fixed part of our experimental design, we chose to fit target question and trial type as fixed effects. Therefore, we fit all models with random intercepts for participant only.

We preregistered a conservative alpha of .01, meaning we consider p-values less than .01 to be statistically significant. We estimate the statistical significance of fixed effects of interest using likelihood ratio tests, comparing a model including the fixed effect of interest to a model excluding it. In addition, we report unstandardized regression coefficients (or odds ratios for logistic regression) with 95% confidence intervals.

First, we tested whether there was evidence for a reuse effect: were people more likely to ask a target-matching question in the exposure condition compared to the no-exposure condition (possibly in interaction with age)? We fit the following logistic regression model (model 1):

*target-matching question* ∼ (exposure condition + previous quality condition + trial type + target question) * age + (1 | participant id)

We initially tested whether there was evidence for an interaction between age and exposure condition. In the absence of a significant interaction, we dropped the interaction between age and exposure condition from the model (retaining all other interactions) and subsequently tested whether there was a main effect of exposure condition. As preregistered, this model only included data from the best, medium, and too-complex trials, as we strongly expected no use of target-matching questions on the worst trial, where the question was completely uninformative.^6^ In the Supplementary Materials, we also report an alternative version of this analysis where we model the number of target-matching questions asked by each participant summed across the four trials.

Next, we tested whether use of a target-matching question in the exposure condition only (i.e., reuse) was affected by the target question’s prior and current informativeness—which were manipulated through previous quality condition (prior informativeness) and trial type (current informativeness). We fit the following logistic regression model (model 2):

*target-matching question* ∼ (previous quality condition + trial type + target question) * age + (1 | participant id)

We initially tested whether there was evidence for interactions between previous quality condition and age and/or between trial type and age. In the absence of significant interactions, we dropped these interaction terms from the model (retaining the interaction between target question and age) and subsequently tested whether there was a main effect of previous quality condition and/or trial type. This model only included data from the exposure condition.

Finally, we adapted both models to investigate recombination. Thus, we fit four additional linear mixed-effects models, using the same predictors as model 1 and model 2 to predict both (1) grammar-based similarity and (2) text-based similarity. Grammar-based similarity was preregistered as a measure of recombination; text-based similarity was not. For the model 1 variants, we used data from all trial types, but only included questions that did *not* match the target question (as recombination only applies for innovated questions). For the model 2 variants, we used data from the exposure condition only, again including only questions that did not match the target question.

As in Study 1, we followed a two-step procedure for all models involving age. We initially fit each of the above models with only children’s data, to estimate the effects of age within childhood. For analyses where there was no evidence for an effect of age within childhood (at the preregistered .01 level), we compare children as a group to adults. For analyses where there was evidence for an effect of age within childhood, we compare 5- to 6-year-olds, 7- to 8-year-olds, 9- to 10-year-olds, and adults. We report only the final regression model from this multi-step analysis.

Because some of our main analyses concern interactions, we use contrast coding for all categorical variables (e.g., for categorical variables with two levels, we code one level as −0.5 and the other level as 0.5). This allows for easy interpretation of all regression coefficients (reported in the Supplementary Materials) even in the presence of interaction terms. Specifically, in all analyses reported below, regression coefficients can be interpreted as the difference between a given condition and the reference group (the first listed condition), holding all other variables fixed at their mean value (i.e., for categorical variables, the average response across all groups). Below, we report only specific statistical tests of interest. Full regression results are included in the Supplementary Materials.

#### Evidence for Reuse.

**Figure 6.**
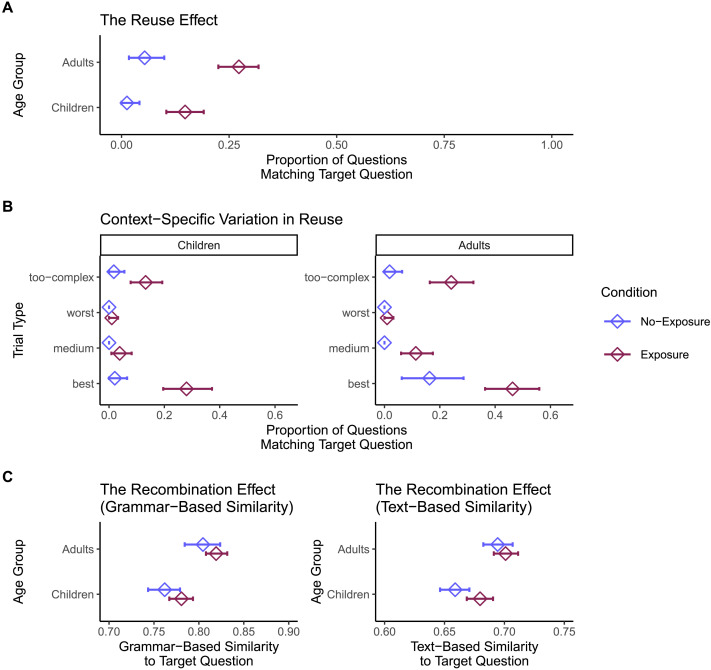
**A:** Frequency of target-matching questions (with bootstrap 95% CIs), in the no-exposure versus exposure condition for children and adults (only for the best, medium, and too-complex trial types, where the target question had informational value). **B:** Frequency of target-matching questions (with bootstrap 95% CIs), across the four trial types for children and adults. *X*-axes truncated for ease of visualization. **C:** Mean similarity (with bootstrap 95% CIs) between asked questions and target question, including only questions that did not match the target question. Left panel uses grammar-based similarity; right panel uses text-based similarity. *X*-axes truncated for ease of visualization.

First, we asked whether there was evidence for reuse. We found greater use of target-matching questions in the exposure condition compared to the no-exposure condition (the “reuse effect”), with no evidence that this reuse effect varied by age (see Figure 6A). Specifically, we fit model 1, with the effect of age group contrasting children and adults. There was no evidence for an interaction between exposure condition and age group, *χ*^2^(1) = 0.51, *p* = 0.48. However, in the absence of the interaction effect, there was evidence for an effect of exposure condition (no-exposure, exposure), *χ*^2^(1) = 55.02, *p* < .001, *OR* = 10.75, 95% CI [4.92, 23.49]. We also estimated the main effect of age group, averaging over all other predictor variables, using estimated marginal means. This revealed a significant effect of age group, *z* = 3.66, *p* < .001, *OR* = 2.93, 95% CI [1.65, 5.22]. To summarize, adults asked the target question more frequently than children overall. However, there was an overall reuse effect, and the magnitude of the reuse effect (i.e., the odds ratio of asking the target question vs. not, for those in the exposure condition compared to the no-exposure condition) did not differ between children and adults.

Next, we investigated *when* participants reused: was reuse more common when the target question was previously and/or currently more informative? We found no evidence that reuse was more common for previously informative questions, but strong evidence that reuse was more likely when the the target question was more informative at the moment of reuse. We fit model 2, with the effect of age group contrasting children and adults. There was no evidence that an interaction between previous quality condition and age group improved model fit, *χ*^2^(1) = 0.004, *p* = .95, nor did the interaction between trial type and age group, *χ*^2^(3) = 0.71, *p* = .87. In addition, in the absence of the interactions, there was no evidence for a main effect of previous quality condition (previously-uninformative, previously-informative), *χ*^2^(1) = 0.38, *p* = .54, *OR* = 0.87, 95% CI [0.55, 1.37]. However, there was evidence for a main effect of trial type, *χ*^2^(3) = 131.09, *p* < .001. As predicted, the probability of asking target-matching questions varied across trial types (see Figure 6B), with higher levels of reuse when the target question would be more informative (e.g., medium compared to best: *OR* = 0.11, 95% CI [0.06, 0.21]) but not overly complex (e.g., too-complex compared to best: *OR* = 0.34, 95% CI [0.21, 0.55]). Thus, to summarize, there was no evidence that people were more likely to reuse the target question when it had been more informative at initial exposure. However, both children and adults were more likely to reuse the target question when it was more informative on the current trial. Moreover, given that asking a target-matching question was rare in the no-exposure condition (especially in the worst, medium, and too-complex trials, where collectively fewer than 1% of questions in the no-exposure condition matched the target question; see Figure 6B), it is likely that this variation across trials reflects context-specific variation in reuse, rather than simply asking good questions independent of previous exposure.

#### Evidence for Recombination.

Next, we asked whether there was evidence for recombination. We found evidence that participants recombined the target question—participants’ innovated (non-reused) questions were more similar to the target question in the exposure condition relative to the no-exposure condition (especially according to text-based similarity). However, this “recombination effect” did not differ in magnitude between children and adults (see Figure 6C). Specifically, we first adapted model 1 to predict grammar-based similarity; the fixed effect for age compared children and adults. There was no evidence for an interaction between exposure condition and age, *χ*^2^(1) = 0.19, *p* = 0.67. However, in the absence of the interaction, questions in the exposure condition were slightly more similar to the target question than questions in the no-exposure condition. This effect did not reach our preregistered .01 significance threshold, *χ*^2^(1) = 5.29, *p* = .02, *b* = 0.02, 95% CI [0.003, 0.03]. However, when we used the same predictors to predict text-based similarity, the results aligned. Again, there was no evidence for an interaction between exposure condition and age, *χ*^2^(1) = 0.73, *p* = 0.39. In the absence of the interaction, there was a significant effect of exposure condition (no-exposure, exposure), *χ*^2^(1) = 7.77, *p* = .005, *b* = 0.02, 95% CI [0.005, 0.03]: participants’ questions were more similar to the target question in the exposure condition relative to the no-exposure condition. Thus, there was an overall “recombination effect”. We also estimated the main effect of age group, averaging over all other predictor variables, using estimated marginal means. This revealed a significant effect of age group. Adults’ questions were overall more similar to the target question for both grammar-based similarity, *t*(352) = 5.46, *p* < .001, Δ*EMM* = 0.04, 95% CI [0.03, 0.06], and text-based similarity, *t*(345) = 4.94, *p* < .001, Δ*EMM* = 0.03, 95% CI [0.02, 0.04]. In sum, while adults’ questions were more similar to the target question than children’s, there was evidence for a recombination effect (as measured by text-based similarity) that did not vary in magnitude between children and adults.

Next, we asked whether recombination varied by the target question’s prior informativeness, adapting model 2 to predict grammar-based similarity and text-based similarity in the exposure condition only. There was no evidence for an interaction between previous quality condition and age group (children, adults) in either the grammar-based similarity model, *χ*^2^(1) = 0.13, *p* = 0.72, or the text-based similarity model, *χ*^2^(1) = 0.12, *p* = 0.73. Moreover, after dropping the interaction between previous quality condition and age group (retaining other interactions), there was no evidence for a main effect of previous quality condition (previously-uninformative, previously-informative)—again, in either the grammar-based similarity model, *χ*^2^(1) = 3.41, *p* = .07, *b* = −0.02, 95% CI [−0.03, 0.001], or the text-based similarity model, *χ*^2^(1) = 0.08, *p* = .78, *b* = 0.002, 95% CI [−0.01, 0.02]. Thus, there was no evidence that recombination was affected by the target question’s prior informativeness. This mirrors the results for reuse: participants’ use of reuse and recombination were both independent of the target question’s prior informativeness during the practice trial.

In an additional exploratory analysis, we also investigated the effect of trial type on recombination in the exposure condition—allowing us to ask whether the degree of recombination varies depending on a target question’s current informativeness. Recall, we had no specific predictions about this effect. However, we found evidence that recombination did vary according to the current informational context. We report these results in the Supplementary Materials.

#### Exploratory: Do Reuse and Recombination Enable Better Questions?

One possible benefit of reuse and recombination is that they may enable the generation of more informative questions at minimal computational cost. Finding informative questions is challenging, especially if good questions are rare. By making certain promising questions (and question components) readily available, exposure to a particular target question may improve later question asking. We tested this possibility in a set of exploratory analyses.

**Figure 7.**
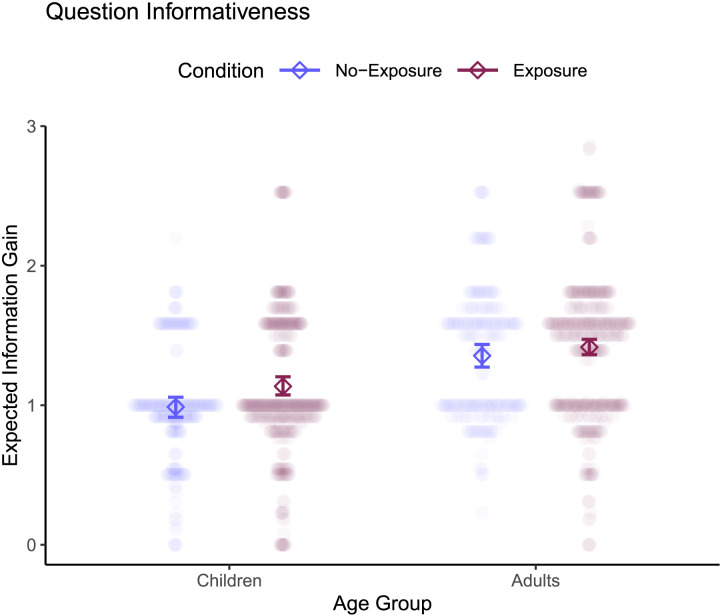
Question informativeness, plotted as a function of age group and exposure condition. Diamonds indicate mean expected information gain with bootstrap 95% confidence intervals, and transparent points indicate the expected information gain of individual questions asked by individual participants.

We found that participants in the exposure condition asked more informative questions on average compared to participants in the no-exposure condition, consistently across ages (see Figure 7). We adapted model 1 to predict question EIG. There was no evidence for an interaction between exposure condition (no-exposure, exposure) and age group (children, adults), *χ*^2^(1) = 2.57, *p* = .11. However, in the absence of the interaction term, there was evidence for an effect of exposure condition, *χ*^2^(1) = 7.59, *p* = .006, *b* = 0.10, 95% CI [0.03, 0.17]. The estimated marginal age group contrast was also significant, *t*(354) = 8.90, *p* < .001, Δ*EMM* = 0.30, 95% CI [0.24, 0.37]. In sum, adults’ questions were more informative than children’s questions overall (unlike in Study 1), and both children and adults in the exposure condition asked more informative questions than those in the no-exposure condition.

This advantage for participants in the exposure condition appeared to be largely driven by reuse rather than recombination. To test this, we added a fixed effect for whether a question matched the target question (non-match, match) to the model above. If the effect of exposure condition on question informativeness was fully driven by reuse, we would expect to see that target-matching questions are more informative than non-target-matching questions, and that the effect of exposure condition is eliminated when controlling for target-matching questions. Indeed, questions that matched the target question were more informative than those that did not, *χ*^2^(1) = 160.28, *p* < .001, *b* = 0.52, 95% CI [0.44, 0.60], and there was no additional effect of exposure condition (no-exposure, exposure) on question informativeness, *χ*^2^(1) = 0.90, *p* = .34, *b* = 0.03, 95% CI [−0.03, 0.10] when controlling for target-matching questions. In other words, the effect of exposure condition on question informativeness was fully explained by reuse. Therefore, reuse—but not recombination—was leveraged to produce better question asking.

#### Exploratory: Reuse and Recombination Across Trials.

Lastly, we replicated the exploratory analyses of reuse and recombination across trials from Study 1. Again, we investigated reuse and recombination on any trial where a given participant asked a valid question on that trial and at least one previous trial. This left 903 trials (446 for children, 457 for adults). For all analyses in this section, mixed-effects models included by-participant random intercepts, with no other random effects. These analyses collapse across all experimental manipulations (e.g., exposure condition).

**Figure 8.**
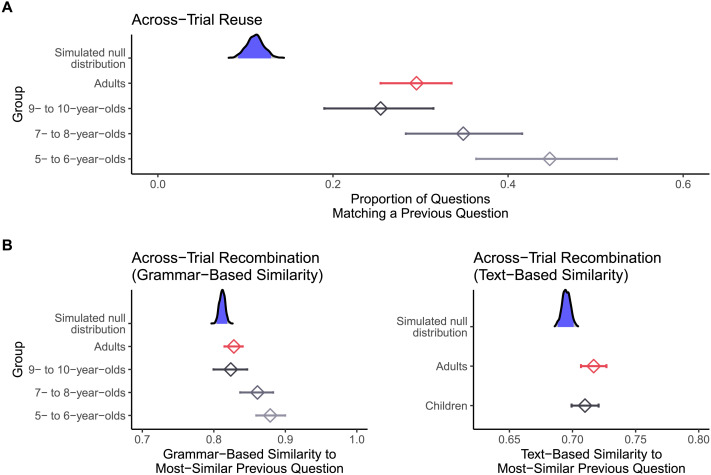
Across-trial reuse (**A**) and recombination (**B**) in Study 2. For all figures, density plots show the simulated null distribution, trial-matched to the true data. The middle 95% of the null distribution is shaded. Diamonds represent the mean observed level of reuse/recombination for each age group, with bootstrap 95% CIs. *X*-axes truncated for ease of visualization.

Replicating Study 1, we found evidence for across-trial reuse in all age groups, with the frequency of reuse decreasing across development (see Figure 8A). In particular, we again found a significant effect of age group (5- to 6-year-olds, 7- to 8-year-olds, 9- to 10-year-olds, adults) on the odds of reusing one’s own earlier question, *χ*^2^(3) = 12.77, *p* = .005. In addition, the amount of reuse in each age group (5- to 6-year-olds, 7- to 8-year-olds, 9- to 10-year-olds, adults) was significantly higher than would be expected by chance based on a simulated null distribution matching the trial structure of the true data (*p*s < .001). These results replicate Study 1, though Study 2 also revealed evidence for developmental change within childhood—perhaps due to the larger sample and thus higher statistical power.

Also replicating Study 1, we found evidence for recombination in both children and adults, with evidence that children’s questions were more similar to their own prior questions as measured by grammar-based similarity, but not text-based similarity (see Figure 8B). Specifically, there was a significant effect of age group (5- to 6-year-olds, 7- to 8-year-olds, 9- to 10-year-olds, adults) on grammar-based similarity between a participant’s question and their most-similar previous question, *χ*^2^(3) = 13.93, *p* = .003. There was no evidence for an age effect (children, adults) on text-based similarity, *χ*^2^(1) = 0.54, *p* = .46, *b* = 0.01, 95% CI [−0.01, 0.03]. However, for both measures, the degree of across-trial recombination was greater than would be expected under a simulated null distribution (*p*s < .01). Thus, there was evidence for across-trial recombination in all age groups, with some evidence that children recombined their own questions more than adults did.

### Discussion

In Study 2, we investigated reuse and recombination in question asking across development, focusing on whether 5- to 10-year-olds and adults reuse and recombine a question from an initial exposure task to a later question asking task. Broadly, we found evidence for reuse and recombination in both children and adults. However, the recombination effect was less robust than the reuse effect, and it was not statistically significant using grammar-based similarity as a measure of recombination. Thus, participants may have asked questions that were semantically related to the target question but did not necessarily use a similar question program representation. In addition, we did not find evidence for developmental differences in reuse and recombination in our main analyses: though adults asked the target question (and other, similar questions) more often than children, this was true in both the exposure condition *and* the no-exposure condition. The difference between conditions, in contrast, did not differ between age groups. Thus, both children and adults reused and recombined prior questions to a similar degree.

Second, we asked under what conditions children and adults reuse and recombine questions. Following prior work on other decision problems (Morris et al., 2021), we investigated two key factors: the extent to which a to-be-reused question was informative in the past, and the extent to which a to-be-reused question was informative in the present. We found that question reuse was strongly sensitive to current informativeness. Further strengthening the conclusion that children and adults reused questions adaptively, participants’ questions in the exposure condition were more informative than questions in the no-exposure condition. That said, we found no evidence that reuse or recombination were more common for previously informative questions compared to previously uninformative questions.

Finally, we replicated the analyses of across-trial reuse and recombination from Study 1. Like Study 1, there was evidence for reuse and recombination across trials: both children and adults reused and recombined their own prior questions. In addition, there was developmental change in trial-to-trial reuse and recombination, with higher rates of reuse and recombination (as measured by grammar-based similarity) in younger children compared to older children and adults.

## GENERAL DISCUSSION

In this work, we investigated two main questions: What is the developmental trajectory of reuse and recombination in question asking? And when do children and adults reuse and recombine questions? We discuss our results on each of these questions in turn.

### What Is the Developmental Trajectory of Reuse and Recombination in Question Asking?

First, we investigated the developmental trajectory of reuse and recombination in 5- to 10-year-olds and adults. We found strong evidence that both children and adults reused prior questions, and this reuse effect could not be fully explained by the informational demands of the task. By creating simulated null distributions for across-trial reuse (Studies 1–2) and including a control, no-exposure condition (Study 2), we found that people reused their own and others’ prior questions above and beyond what would be expected based on trial structure alone. Thus, reuse seems to reflect a specific effect of past questions on later question asking.

We also found some evidence for recombination, though the evidence was more variable. Studies 1 and 2 found that children and adults recombined their own prior questions, reflected in their tendency to ask questions that were highly similar to their own prior questions (using both grammar- and text-based similarity measures). In Study 2, however, when we experimentally manipulated exposure to particular questions, children and adults recombined prior questions when measured using a general measure of semantic similarity (text-based similarity), but not a measure that assumes a specific grammar-based recombination process (grammar-based similarity). Thus, participants may have asked questions that were semantically related to the target question, but without necessarily using a similar question program representation. Moreover, effect sizes were generally weaker for the recombination effect compared to the reuse effect. In sum, there was some evidence for recombination, but it was a smaller effect.

We found mixed evidence for developmental change in reuse and recombination. In both Study 1 and Study 2, children reused their own prior questions at higher rates than adults. When operationalizing recombination using grammar-based similarity, children recombined their own prior questions more than adults. That said, there was no evidence for developmental change in reuse and recombination from an exposure phase to later question asking in Study 2, and there was no evidence that children recombined their own prior questions more than adults when recombination was measured using text-based similarity.

Based on these results, children do not appear to be *more* exploratory than adults in their tendency to stray from prior questions—in some cases, children were *less* exploratory in their question asking, preferring to reuse and recombine their own prior questions more frequently than adults. This could reflect that question asking is a challenging search problem, and reuse and recombination provide a simpler alternative to searching the space of possible questions. Children, who have more limited computational resources than adults, may be especially likely to rely on simple shortcuts when solving challenging problems. In this case, this results in less “exploratory” question asking. That said, it is possible that children’s question asking is exploratory in other ways, akin to other kinds of search (Gopnik, 2020).

### When Do Children and Adults Reuse and Recombine Questions?

We also investigated the context-specificity of reuse and recombination: are children and adults more likely to reuse and recombine questions when those questions (1) were more informative in the past, and (2) are more informative in the present?

We found that question reuse was strongly sensitive to current informativeness across development. This is important because engaging in too much reuse could have informational costs: repeating questions from the past without reflection about one’s present situation could lead to asking uninformative questions (e.g., asking “How many monsters have a square head?” when all head shapes are known). Instead, children and adults alike were more likely to ask the target question when it was more, compared to less, informative. In addition, the exposure manipulation in Study 2 increased the informativeness of participants’ questions, suggesting that reuse was leveraged to produce informative questions. We did not have specific predictions about how recombination would vary according to current informativeness, but we reported exploratory analyses on this question in the Supplementary Materials.

We also asked whether reuse and recombination were stronger for questions that had previously been more informative. We did not find evidence for this prediction. Thus, though prior quality is instrumental in shaping what comes to mind in other contexts (see Morris et al., 2021), this may not hold for question asking. That said, it is possible that our manipulation of past informativeness did not affect later question asking because a single exposure does not provide sufficient evidence for a question’s general quality. In Morris et al. (2021) and other related work (e.g., Bear et al., 2020), the “previous quality” of an option was manipulated by pairing it with a signal of high quality over a large number of training trials. In the present research, we were constrained by children’s limited attention span for online studies, so we included only one exposure trial. However, in future research, it would be valuable to investigate whether additional exposures would increase the influence of a question’s prior informativeness on later question asking.

### Open Questions

Based on the evidence presented here, we cannot conclusively explain why we found mixed evidence for developmental change in reuse. Specifically, we found that children reused their own prior questions more than adults, but there was no age difference in reusing a target question provided by a confederate. One possibility is that the decision to reuse is partly motivated by how costly (e.g., cognitively effortful) reuse is expected to be—and these costs vary across both situations and development. For example, it may be more costly for children to reuse *others’* questions (especially given that the questions used in Study 2 were rather complex), compared to *their own* questions. If this difference in costs is stronger for children than adults, it could explain the mixed developmental patterns.

Indeed, one limitation of this study is the complexity of the target questions used in Study 2. While children were capable of generating accurate answers to these questions (see Supplementary Materials), actually using a provided answer to identify the hidden monsters may be challenging, especially for young children. For example, a child might know that the red monster has two legs and the purple monster has one leg, and they might learn from the question “How many legs do all the monsters have combined together?” that there are six total legs. However, correctly identifying that the blue monster has three legs requires arithmetic that could be beyond children’s capacities. This could explain why the target questions used in Study 2 were rarely asked in the no-exposure condition, especially in childhood (see Cheyette et al., 2023). Moreover, even when children *do* use these questions, their likely inability to accurately interpret the answer might limit the extent to which these questions are actually informative for children. Thus, it would be useful for future work to investigate a broader range of questions as possible targets for reuse and recombination.

Our results are also ambiguous as to *why* people are less likely to reuse questions when they are less informative in the current situation. One possibility is that a previous question comes to mind regardless of its current informativeness, then uninformative questions are eliminated from consideration. Another possibility is that only currently informative questions come to mind—meaning a previous question will not even be considered when it is uninformative.

There are two reasons to favor the former account. First, prior work has shown that previous solutions (e.g., possible dinner ideas) come to mind independently of their current quality, then high-quality solutions are selected and low-quality solutions are discarded (Morris et al., 2021). If the process by which one finds a question from a large space of possible questions resembles the process by which one finds a solution from a large space of possible solutions, we would expect question asking to follow this same pattern. Second, our Study 2 provides indirect evidence that previous questions come to mind regardless of their current informativeness. If a previous question that is currently uninformative fails to come to mind, we might expect to see weaker evidence for recombination in this case. This is because there would be no previous question in mind on which to anchor the search for novel questions. In the Supplementary Materials, we report evidence against this possibility. While this evidence is suggestive, further research is needed to fully uncover the cognitive processes underlying context-specificity in reuse.

Relatedly, it is unclear from the present studies whether our results reflect that participants *learned* which questions to ask, as opposed to simply being *primed* to think of particular questions. We suspect that both priming and learning underlie reuse and recombination, perhaps at different stages of the learning process. At first, children might reuse and recombine others’ questions (or their own questions) due to simple priming. For example, while reading a picture book, a parent might ask their child “What sound does a cow make?” Then, two pages later, looking at a picture of a pig, the child might reuse this question, asking “What sound does a pig make?” This may be explained by priming. However, we suspect that real-world reuse and recombination go beyond priming. For example, several weeks later, the child might ask “What sound does an ostrich make?” when speaking to their aunt at a zoo. In this case, the question-asking situations are separated in time and context, increasing the likelihood that the child genuinely learned that it is useful to ask what sound an animal makes (rather than being merely primed to think of this question).

Because we investigated reuse and recombination in a single, 15-minute experimental session, it is not clear whether previous questions affected participants’ later questions due to priming, learning, or both. Notably, our results cannot be fully explained by priming because participants selectively reused questions in situations where it was more informative to do so. Thus, at the very least, these results reflect that children and adults can override primed questions depending on the informational context. However, additional research is needed to experimentally test whether children and adults can genuinely *learn* which questions are likely to be reasonable in a given context, then reuse and recombine those learned questions across situations.

Relatedly, while we presented evidence for reuse and recombination in “near transfer” situations (across trials of the same question asking task), many real-world instances of reuse and recombination could require “far transfer” (see Barnett & Ceci, 2002). For example, in the realistic episode described above, the learner has to recognize that the present situation—trying to learn about the ostrich at the zoo—has commonalities to some prior situation—reading a book about a cow at home. Generalizing questions across disparate situations in this manner is likely to be cognitively demanding. Indeed, research has shown that spontaneously recognizing analogies across situations can be difficult (Gick & Holyoak, 1980). If reusing and recombining questions requires recognizing explicit connections between disparate situations, we might expect lower levels of reuse and recombination in real-world contexts compared to our experimental context. However, we would still expect that reuse and recombination occur when such connections across situations are made.

Lastly, real-world reuse and recombination require broader generalization across question “parameters.” In our task, participants repeatedly seek information about monsters that have the same colors, possible head shapes, and possible numbers of legs. In the real world, there is far broader variation in such parameters: for example, one can learn about millions of animal species, and the number of possible animal sounds is likely impossible to enumerate. The complexity of these real-world question asking situations might make reuse and recombination even more powerful strategies: the more possible parameters there are, the more difficult it might be to find a reasonable question in any particular situation. However, further research is needed to extend our work on reuse and recombination to these more realistic contexts.

### Implications for Understanding Question Asking and Search

Our results also have implications for future research on both question asking and other search problems.

#### Question Asking.

Understanding the cognitive mechanisms underlying question asking has theoretical import for fleshing out our notion of the “child as scientist” (Gopnik & Wellman, 2012). Amidst evidence that very young children are effective causal learners (Gopnik et al., 2001; Kushnir & Gopnik, 2005; Meltzoff et al., 2012; Schulz et al., 2007; Sobel et al., 2007), theory-builders (Bonawitz et al., 2019; Carey, 1985; Keil, 1992; Wellman & Gelman, 1992), and explorers (Bonawitz et al., 2012; Cook et al., 2011; Lapidow & Walker, 2020; Schulz & Bonawitz, 2007), children’s failure to generate informative questions in standard 20-questions tasks stands out as a puzzling result. Prior work has appealed to the linguistic requirements of the task (Swaboda et al., 2022), low levels of cognitive flexibility in young children (Legare et al., 2013), the challenge of identifying abstract categories to query (Ruggeri & Feufel, 2015; Ruggeri et al., 2019, 2021), and the challenge of avoiding redundant questions (Chai et al., 2023; Ruggeri et al., 2016).

Surprisingly, we found no evidence for a difference in question informativeness between children and adults in Study 1, and only a small difference in Study 2. Prior work has documented a large improvement in the ability to identify and generate informative questions between ages five and ten, with only ten-year-olds achieving adult-like performance (Jones et al., 2020). We speculate that children’s superior performance in the present research can be attributed to two specific features of our task. First, prior work has shown that one source of young children’s inefficient question asking is their tendency to continue question asking beyond the point at which no uncertainty remains (Chai et al., 2023; Ruggeri et al., 2016). In our task, this is impossible because participants only ask one question per trial. Second, prior work has shown that children often rely on suboptimal “hypothesis-scanning” questions: questions that rule out or confirm a single hypothesis, rather than a larger subset of hypotheses (Herwig, 1982; Mosher & Hornsby, 1966). In our task, each hypothesis is a conjunction of six features across three monsters. Thus, asking a hypothesis-scanning question is difficult: “Does the red monster have a square head and two legs, the blue monster a circle head and one leg, and the purple monster a circle head and three legs?” Interestingly, some children in our studies did ask a hypothesis-scanning question of this sort. However, the difficulty of formulating such questions may have pushed children towards asking more informative questions in general, compared to prior work. That said, both adults and children asked less informative questions than were theoretically possible in our task (see Study 1). This could be because people care about particular features of questions that go beyond their informativeness: for example, their length (Rothe et al., 2017) or the interpretability of their answers (Cheyette et al., 2023).

Our work also points towards a novel limitation on informative question asking in both children and adults: asking good questions may be more difficult when one lacks a “store” of relevant prior questions to reuse or recombine. This may account for children’s apparent success asking questions in real-world settings (e.g., Callanan & Oakes, 1992; Greif et al., 2006), where a rich store of relevant prior questions is available. This rich store of prior questions (asked by oneself and by others) might be key to producing “scientist-like” question asking (or perhaps “hacker-like” question asking, see Rule et al., 2020).

Future work could build on these insights by investigating variation in question asking across domains, situations, or individuals. Question asking has largely been viewed in prior research as a domain-general capacity, which can be exercised in a diverse range of situations. There is surely something right about this view—regardless of the situation, question asking requires (1) identifying a source of uncertainty, (2) formulating a question that is likely to resolve that uncertainty, (3) directing that question to a knowledgeable individual, and (4) evaluating the received response (Mills & Sands, 2020; Ronfard et al., 2018). However, question asking is also constrained by an individual’s domain-specific experience and knowledge. Reuse and recombination are one manifestation of this influence of prior experience. Additional research is needed to investigate other similar constraints.

This work also raises new questions about the representations and algorithms underlying question asking. In this work, we used two measures of recombination: Grammar-based similarity is a narrower measure of recombination, which assumes that questions are represented in a computational language of thought and generated by (1) starting from a previously-used question and (2) making incremental edits to it. Text-based similarity is a broader measure of recombination that encompasses any semantic similarity between previous questions and new questions (measured using black-box machine learning methods).

Results with the broader measure of recombination were more consistent with our predictions: in Study 2, the effect of prior exposure on text-based similarity was significant, and the effect of prior exposure on grammar-based similarity was not. Moreover, adults’ similarity judgments were strongly related to text-based similarity, but unrelated to grammar-based similarity (see Supplementary Materials). This does not necessarily imply that the representations and algorithms underlying human question asking are more like a black-box machine learning model than a computational grammar, though this could be the case. The results are compatible with a number of possibilities. For example, people might use a grammar-like representation to generate questions, but with an a different algorithm than that assumed by our grammar-based similarity measure (see Cheyette & Piantadosi, 2017; Zhao et al., 2024, for other possible grammar-based algorithms). Alternatively, people might use the assumed grammar-like representation *and* the assumed algorithm—but with a different grammar than our task-specific language (see Piantadosi et al., 2016). In a different grammar, grammar-based similarity would likely produce different results, while text-based similarity does not rely on any particular grammar.

Thus, while our results show that reuse and recombination are mechanisms underlying question asking, the representations and algorithms underlying reuse and recombination remain unclear. Future work is needed to systematically compare and evaluate different possible mechanisms that underlie how humans generate, reuse, and recombine questions.

#### Search Problems.

Finally, this work has implications for understanding other problems that require searching through large possibility spaces: for example, deciding what to cook for dinner, what to name a pet, or how to solve a problem. First, it is notable that we find evidence for reuse and recombination in a new domain: question asking. This suggests that various search problems—that are quite disparate on the surface—might tap into the same underlying cognitive mechanisms.

That said, there may be some differences between different kinds of search problems. Though prior work has shown that reuse is more common for solutions that were previously higher-quality (Bear et al., 2020; Morris et al., 2021; Phillips et al., 2019), we did not find evidence for this phenomenon in question asking. As mentioned above, this could have been because participants failed to form a belief about a question’s general quality from a single exposure. On the other hand, ignoring previous quality could be adaptive if the correlation between past and present quality is lower for question asking than for other decision tasks. For example, the dinner recipes that have been good in the past will typically remain good in the present—and events that could change this correlation are infrequent (e.g., becoming a vegetarian). For question asking, in contrast, the situations we face are ever changing, and our knowledge can change with each question we ask. Thus, it may be rare for previously informative questions to remain informative in the present. In future work, it may be fruitful to investigate whether people’s sensitivity to past quality—in various kinds of search problems—depends on learned correlations between past and present quality.

The present research also raises new questions about how to define and measure reuse and recombination—which is also relevant for studying other search problems. For example, most prior investigations of recombination in the domain of hypothesis search (e.g., Gelpi et al., 2020; Herbst et al., 2017; Zhao et al., 2024) have measured recombination in a binary way (a hypothesis either recombines some key component of a prior hypothesis, or it does not). In contrast, we operationalize recombination using continuous measures of semantic similarity. As a result, our work provides new insight into how the *degree* of recombination varies across ages and situations.

That said, the ideal measure for the degree of recombination remains unclear. As discussed above, the present work used two measures of recombination: grammar-based similarity and text-based similarity. While results were sometimes consistent between these two measures, there were some discrepancies. Further work is needed to fully understand why we found differences between grammar-based and text-based measures of similarity in the present research. As noted previously, text-based similarity might capture a broader range of possible representations and algorithms underlying recombination. At the same time, text-based similarity could be *too* broad—two questions could be semantically similar (or identical) without necessarily resulting from a recombination-like generation process. Thus, text-based similarity could provide an over-estimation of “actual” recombination. Developing the most accurate possible measure of recombination is beyond the scope of the current work, but we hope that future research will take up this challenge, further uncovering the mechanisms underlying recombination.

Relatedly, what defines a “template” for reuse? In the present research, we defined a question template as a program representation that uses a particular set of functions in a particular configuration, but optionally allows different parameters of the same type. More concretely, the questions “Does the blue monster have two legs?” and “Does the red monster have three legs?” are instances of the same question template because their program representations—(= = (legs Red) 3) and (= = (legs Blue)2)—take the same form and use the same functions. But why can two questions with different arguments be counted as instances of the same question template, but two questions with different functions (e.g., (= = (legs Red) 3) and (= = (shape Red) Square)) cannot? We defined reuse this way because functions correspond to meaningful features in our task—asking about head shape is asking about a fundamentally different thing than asking about number of legs. However, it is possible that question templates (and therefore reuse) can be conceptualized at multiple levels of abstraction: two questions that share the same functions use the same “concrete” template, while two questions that use different functions in the same configuration use the same “abstract” template. Moreover, reuse can be even more narrow: sometimes people reuse the exact same question, as opposed to a new question with the same template (both were counted as reuse in the current research, and we did not analyze them separately). Specifying how people reuse at multiple levels of abstraction would be a promising direction for future work.

Finally, are reuse and recombination really distinct? One way of viewing reuse is as an extreme case of recombination, where *all* (rather than some) components of a prior question are repurposed in a new situation. Our results for reuse and recombination were largely similar, in both developmental trajectory and contextual moderators. Thus, our work is consistent with the possibility that reuse is continuous with recombination. However, understanding the precise relation between reuse and recombination would require additional investigation of the underlying cognitive processes (e.g., memory, search). Again, we hope that future research across a range of search problems can provide further insight.

### Conclusion

Taken together, this research provides evidence that 5- to 10-year-olds and adults reuse and recombine previous questions. They do so in ways that are sensitive to the informational context, reusing most when it is most informative to do so. In addition, in some cases, children are more likely to reuse and recombine prior questions than adults. This work provides new insight into the mechanisms behind question asking and, more broadly, has implications for understanding how humans find good options in a large space of possibilities—whether solving problems, generating hypotheses, or asking questions to learn.

## ACKNOWLEDGMENTS

We would like to thank Brenda Chavez-Olguin, Sophia Cordeiro, Alison De Leon, Julia Johnson, Megan Moran, Archisha Murthy, Jasmine Tepper, and Barron Tsai for their assistance with methodology, data collection, and data processing. We are grateful to Anselm Rothe and Ziyun Wang, whose modeling code for the Battleship task was adapted to our new task. We are also grateful to members of the Computation and Cognition Lab and the Conceptual Development and Social Cognition Lab at New York University for their valuable feedback on this work. Finally, some of the results reported here were presented at the 2024 meeting of the Cognitive Science Society, and we are grateful to this audience for their discussion and feedback.

## FUNDING INFORMATION

This work was supported by a National Science Foundation SBE Postdoctoral Research Fellowship to E.G.L. under grant no. 2204021 and National Science Foundation grant no. 2000617 to M.R.

## AUTHOR CONTRIBUTIONS

Emily G. Liquin: Conceptualization; Data curation; Formal analysis; Funding acquisition; Investigation; Methodology; Project administration; Software; Validation; Visualization; Writing – original draft; Writing – review & editing. Marjorie Rhodes: Conceptualization; Funding acquisition; Methodology; Resources; Supervision; Writing – review & editing. Todd M. Gureckis: Conceptualization; Funding acquisition; Methodology; Resources; Software; Supervision; Writing – review & editing.

## DATA AVAILABILITY STATEMENT

Further methodological details can be found at https://osf.io/r3usj/. Data and analysis scripts can be found at https://github.com/emilyliquin/question-reuse.

## Notes

^1^ There are surely other difficulties, several of which have been studied in prior work. For example, children also sometimes struggle to decide who is best suited to answer any particular question (Mills et al., 2010, 2011), and adults weigh the desire for simple answers against the desire to gain information (Cheyette et al., 2023).^2^ Specifically, rather than using participants’ actual questions as input to the machine learning model, we created “standardized” natural language versions of each question based on the question’s program representation. For example, all questions translated as (= = (shape Red) Square) were assigned the standardized question “Does the red monster have a square head?”, regardless of how a participant actually expressed that question. We did this because we suspected that children might use more idiosyncratic phrasing to express the same ideas as adults. Standardizing similarity to be solely a function of question meaning removes the influence of these idiosyncratic differences in phrasing. Note that adult participants judged similarity between the original questions as asked—but text-based similarity was computed over standardized questions.^3^ There are several other ways of formalizing question quality (Hawkins & Goodman, 2017; Nelson, 2005; Nelson et al., 2014). We used EIG in the present research to facilitate comparisons to other work on children’s question asking (e.g., Meder et al., 2019; Nelson et al., 2014; Ruggeri et al., 2016, 2017; Török et al., 2024), and because an existing Python implementation of EIG for Rothe et al.’s (2017) Battleship Task (https://github.com/anselmrothe/EIG) could be easily adapted to our task.^4^ Note that this “maximally informative” question, which is guaranteed to resolve all uncertainty, may not be feasibly expressed in the task-specific language or in natural language.^5^ In the Supplementary Material, we test whether participants generally answered the confederate’s question correctly. If they did not, it would suggest that participants could not understand the target question. In contrast, most participants selected a correct answer, even the youngest children (82% of 5- to 6-year-olds). Moreover, there was no evidence that the prevalence of correct answers differed between the two target questions. This suggests that it is reasonable to assume that most participants across our age range have the capacity to understand these target questions.^6^ In fact, across the full sample there were only two instances of target-matching questions on this trial (one 9-year-old and one adult, both in the exposure condition).

## Supplementary Material


