# Supplementary material for: Seeking New Information With Old Questions: Children and Adults Reuse and Recombine Concepts From Prior Questions
Source: Open Mind (Camb). 2025 Jul 26;9:885–925. doi: 10.1162/opmi.a.12 (PMC12373455; doi:10.1162/opmi.a.12)
Supplement: Supplementary file 1 [file opmi-09-885-s001.pdf]

Supplementary materials for “Seeking new information with old questions: Children and adults reuse and recombine concepts from prior questions”

Emily G. Liquin, Marjorie Rhodes, and Todd M. Gureckis

Task-Specific Language

We developed a task-specific language to capture question asking in our task, heavily based on Rothe et al.’s (2017) task-specific language for question asking in the Battleship task.

The task-specific language is structured as a grammar with a set of rewrite rules. Every valid expression is interpreted as a question. All valid expressions start with symbol *A* (for “answer"). Expressions are then expanded via recursive rewrites, until no further rewrite rules apply. The full grammar is included in Table 1.

Table 1

Grammar for producing question program representations.

**Answer types**	
A → B	*Boolean*
A → N	*Number*
A → S	*Shape*
**Booleans (yes/no)**	
B → True	
B → False	
B → (not B)	
B → (and B B)	
B → (or B B)	
B → (== B B)	
B → (== N N)	
B → (== S S)	
B → (=== setN)	*True if all elements in set of numbers are equal*
B → (=== setS)	*True if all elements in set of shapes are equal*
B → (any setB)	*True if any element in set of booleans is true*
B → (all setB)	*True if all elements in set of booleans are true*

B → (> N N)
B → (< N N)

Numbers

N → 0	
...	
N → 9	
N → (+ N N)	
N → (+ B B)	
N → (++ setN)	*Sum of a set of numbers*
N → (++ setB)	*Number of true elements in a set of booleans*
N → (- N N)	
N → (legs M)	*Number of legs of monster M*
N → (setSize setM)	*Number of elements in a set of monsters*
N → (setSize setN)	*Number of elements in a set of numbers*
N → (setSize setS)	*Number of elements in a set of shapes*

Shapes

S → Square	
S → Circle	
S → (shape M)	*Head shape of monster M*

Monsters

M → Blue	*The blue monster*
M → Red	*The red monster*
M → Purple	*The purple monster*

Mapping

setB → (map fxB setM)	*Map a boolean expression onto a set of monsters*
setN → (map fxN setM)	*Map a numeric expression onto a set of monsters*
setS → (map fxS setM)	*Map a shape expression onto a set of monsters*

Lambda Expressions

fxB → (λ x B)	*Boolean expression with monster variable*
fxN → (λ x N)	*Numeric expression with monster variable*
fxS → (λ x S)	*Shape expression with monster variable*

Sets

setM → (shapeObjects S)	*Set of all monsters with head shape S*
setM → (legsObjects N)	*Set of all monsters with number of legs N*
setM → (union setM setM)	*Combine two sets of monsters*
setM → (intersection setM setM)	*Elements that exist in both sets of monsters*

setM → (unique setM)	*Unique monsters in set*

Determining a Measure of Recombination: Question Similarity

We formulated several possible measures of question similarity, which we used as proxies for measuring the degree of recombination. We describe each measure in more detail in the following paragraphs. Then we report a study that we conducted to select a measure of similarity for our primary analyses.

Candidate measures of recombination

Tree edit distance (i.e., grammar-based similarity)

Question program representations can naturally be represented as trees. Each function or argument in a program is a node in the tree. Arguments are the children of their parent functions. Functions can also be nested within functions. For example, the question "Does the blue monster have a circle head?", or `(== (shape Blue) Circle)`, can be represented with the following tree:

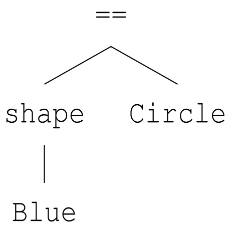

We computed the tree edit distance between two programs using the Zhang-Shasha algorithm (Zhang & Shasha, 1989). This algorithm computes the minimum number of edits (insertions, deletions, or relabelings) needed to go from one tree structure to another. Argument nodes of the same type (i.e., two numbers) were treated as equivalent, so that the tree edit distance between two questions that used the same "question template" was zero.

Tree edit distance is a reverse-scored measure of similarity: questions that are more distant from each other are less similar. To facilitate comparisons with other similarity measures, we norm tree edit distance for each distance d_i of all distances d as follows: $1 - (d_i/max(d))$. Thus, a grammar-based similarity score of 1 indicates a tree edit distance of 0. A grammar-based similarity score of 0 indicates the maximal tree edit distance represented in the data.

Text-based similarity (original and standardized)

We computed text similarity between two natural language questions using the following procedure. Each question was converted to a 384-dimensional vector using pre-trained Sentence-BERT embedddings (all-MiniLM-L6-v2 model; Reimers & Gurevych, 2019). Then, we computed cosine similarity between the resulting question vectors. We

used Sentence-BERT because it represents the meaning of full sentences (e.g., questions). As a result, Sentence-BERT is faster and more accurate at computing sentence embeddings, compared to word-level models (Reimers & Gurevych, 2019). We used the all-Mini-LM-v2 pretrained embedding model because the Sentence Transformers Python documentation describes it as a general-purpose model with both good quality and fast performance.

We developed two possible variants of this procedure, which pre-processed participants' questions to different degrees. For the "original questions" variant, we removed all filler words (e.g., "like," "um"). In addition, we removed all pre- or post-question speech from children's questions. For both adults' and children's questions, we ensured that every question started with a capital letter and ended with a question mark, and that all numbers were expressed in words (rather than numerals). Otherwise, questions were not edited prior to being projected to the embedding space.

For the "standardized questions" variant, we used the question program representations to produce a single standardized natural language question for each unique program representation. We did this to partial out possible age-related differences in phrasing and grammar. Using the program representations, the first author back translated each unique program representation into a natural language question—prioritizing as much consistency as possible across the full set of questions (e.g., always referring to the monsters and features in the exact same way). For example, all questions translated as `(== (shape Red) Square)` were assigned the standardized question "Does the red monster have a square head?", regardless of how a participant actually expressed that question (e.g., "Is the red monster's head shape a square?"). These standardized questions were then projected into the embedding space.

Shared functions and shared arguments

We calculated the proportion of functions in one question's program representation (e.g., ===, `shape`) that were also present in a second question's program representation. We also calculated the proportion of arguments in one question's program representation (e.g., `Blue`, `Square`) that were also present in a second question's program representation. For questions with lambda expressions, we filtered out `lambda` and `x0` prior to calculating these measures.

Note that these measures of similarity are asymmetric, as they depend on the total number of functions or arguments in a question. As an intuitive example, 100% of the functions in the question `(== (shape Blue) Circle)` are present in the question `(++ (map (lambda x0 (== (shape x0) Square)) (set b r p)))`, but the latter question has several additional functions that are not present in the former question. For recombination, we define similarity as the proportion of functions/arguments from the *second* (later in time) question that come from the *first* (earlier in time) question.

Method

To select a similarity measure to use for our primary analyses of recombination, we compared the predictions of each of the above measures to adult similarity judgments.

Participants

We recruited a sample of 110 adult participants from Prolific (ages 20 to 71, 59% male, 39% female, and 2% non-binary or genderqueer). An additional 3 participants were excluded from analyses for failing to pass two simple attention checks (selecting a specified scale option on a multiple-choice question, identifying the contents of a picture).

Materials

Each participant rated similarity between 15 pairs of questions, drawn randomly from a larger set of 100 question pairs. The 200 questions that comprised these 100 question pairs (plus 12 additional questions for instructions) were randomly drawn from participants' questions in Studies 1 and 2 (53 questions per age group per study).

Procedure

Participants were provided a basic description of the question asking task, including an introduction to the monsters and their features. They were shown 12 example questions to facilitate their understanding of the task.

Then, participants were told that they would be judging the similarity between multiple questions. Participants were provided the following instructions:

> As you saw on the previous screen, the questions vary in many ways: what they're asking about, how complex they are, their sentence structure, and so on. **Please do your best to judge the *overall* similarity between the questions**, taking all of these dimensions into account.
>
> All of the questions were asked to figure out which monsters were hidden in the box. In this sense, all the questions are similar, because they ask about the monsters in the box. Please **do not** take this into account when judging similarity. We want you to judge the similarity of questions within the context of this task. Some questions might be considered quite dissimilar, even though both questions ask about monsters.

Participants were given examples of two highly similar questions ("How many square heads are in the box?" and "How many square heads are there?") and two highly dissimilar questions ("Do the red one have a square head and the purple one have a circle head and the blue one have a square head?" and "How many legs does the purple monster have?"). Participants were instructed to ignore things like grammar, typos, or the participant's likely age.

Participants completed "practice" similarity ratings on four pairs of questions, in order to calibrate all participants' similarity ratings to the same initial set of questions. Similarity ratings were made on a six-point scale (extremely dissimilar, dissimilar, somewhat dissimilar, somewhat similar, similar, extremely similar).

Finally, participants rated the similarity between 15 target pairs of questions, drawn at random from the larger set of 100 question pairs. Within each pair, the two questions were presented in a randomized order for each participant.

Table 2

Correlations between similarity measures and human judgments. For shared functions and shared arguments, each correlation corresponds to one possible ordering of questions within each pair.

Measure	Description	Correlation with human judgments
Grammar-based similarity	Number of edits required to get from one question's program representation to another question's program representation (normed so that higher similarities correspond to smaller numbers of edits)	0.11
Standardized text-based similarity	Cosine similarity between question embeddings (with each question standardized so that each program representation is associated with a single unique natural language question)	0.88***
Original text-based similarity	Cosine similarity between question embeddings (using the original asked question for each question)	0.65***
Shared functions	Proportion of functions in one question's program representation that are also in the other question's program representation	0.60*** / 0.58***
Shared arguments	Proportion of arguments in one question's program representation that are also in another question's program representation	0.23* / 0.10

Note: $^{*}p<0.05$; $^{**}p<0.01$; $^{***}p<0.001$

Results and Discussion

For each of the 100 question pairs, we computed grammar-based similarity, standardized text-based similarity, original text-based similarity, shared functions, and shared arguments. Because the order of questions in each pair was randomized, there was no natural definition of the "first" and "second" question for asymmetric similarity measures (shared functions and shared arguments). As a result, we computed these measures for both possible question orders. This resulted in a total of 7 similarity measures.

For each of the 100 question pairs, we computed the mean human-rated similarity. Then, we correlated the mean human-rated similarity with each similarity measure. The results are reported in Table 2 and Fig. 1.

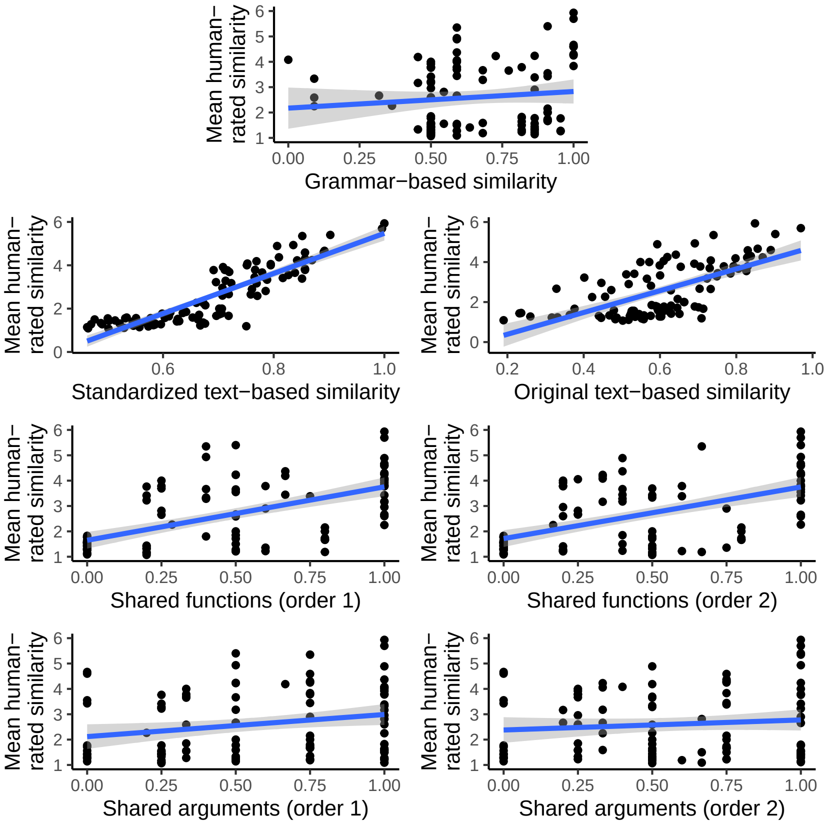

Figure 1

Associations between each similarity measure and human judgments. Each point corresponds to one question pair.

The correlation between standardized text-based similarity and human-rated similarity was very strong ($r(98) = 0.88$). Human-rated similarity was also correlated with original text-based similarity and shared functions, but more weakly.[1]

On the basis of these results, we conducted all analyses using two measures of question similarity: standardized text-based similarity (which was most strongly related to human similarity judgments) and grammar-based similarity (which was more interpretable using our question grammar and was preregistered for Study 2).

Formalizing Question Informativeness

To measure question informativeness, we used expected information gain (EIG; Lindley, 1956; Oaksford & Chater, 1994). This measure has frequently been used in prior

[1]Combining shared functions and shared arguments into an overall “shared program elements" score also did not produce a stronger correlation with human similarity judgments: $r(98) = 0.50$ for one question ordering and $r(98) = 0.43$ for the other question ordering.

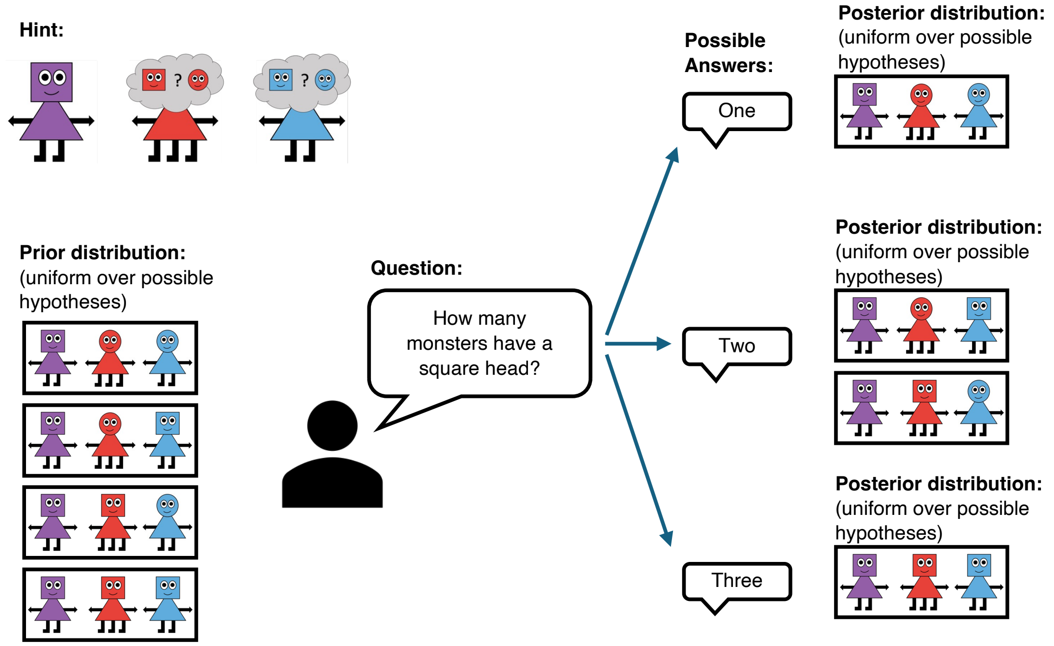

Figure 2

Illustration of hypothesis space updates from hint to answer.

work on question asking (e.g., Liquin & Gureckis, 2022; Rothe et al., 2018; Ruggeri et al., 2016).

EIG describes the extent to which a question is likely to reduce uncertainty. Uncertainty is a property of a *hypothesis space*: a set of mutually exclusive possibilities, only one of which is true. In our task, each hypothesis is a set of monsters: one blue monster, one red monster, and one purple monster, each with a particular head shape and number of legs.

The "hint" provided on a given trial constrains the hypothesis space to only the sets of monsters consistent with the revealed features. For example, Fig. 2 depicts all hypotheses consistent with a hint in which two monsters' heads are covered: both monsters' heads could be circle, both monsters' heads could be square, or there could be one of each shape. We assume that the prior distribution $p(h)$ is uniform over all possible hypotheses consistent with the hint.

The informativeness of a question is defined by the informativeness of its possible answers. For any given question, q, receiving answer d allows one to update the hypothesis space. In particular, the posterior distribution is computed using Bayes' rule,

$$p(h|d;q) = \frac{p(d|q;h)p(h)}{\sum_{h' \in H} p(d|q;h')p(h')}. \tag{1}$$

We assume that the likelihood $p(d|q;h')$ is 1 if answer d to question q is consistent

with the true hypothesis h and 0 otherwise. As a result, the posterior distribution $p(h|d;q)$ is uniform over all possible hypotheses consistent with both the hint and the answer (see Fig. 2).

Intuitively, a good answer reduces our uncertainty about the world. Formally, the Information Gain (IG) associated with an answer to a particular question is defined

$$IG(d;q) = I[p(h)] - I[p(h|d;q)], \tag{2}$$

where $I[p(h)]$ is the Shannon entropy (i.e., uncertainty; Shannon, 1948) of the prior distribution and $I[p(h|d;q)]$ is the Shannon entropy of the posterior distribution. Thus, the informativeness of an answer is the degree to which it reduces uncertainty about the true hypothesis. In our task, entropy is a function of the size of the hypothesis space. In Fig. 2, the answers "one" or "three" will reduce Shannon entropy from 2 to 0 (IG = 2), while the answer "two" will reduce Shannon entropy from 2 to 1 (IG = 1).

To define the informativeness of a *question*, we must account for all its possible answers. Therefore, the Expected Information Gain (EIG; Lindley, 1956; Oaksford & Chater, 1994) of a question is determined by the Information Gain of each answer, weighted by the answers' probability:

$$EIG(q) = \sum_{d \in A_q} p(d|q) IG(d;q) \tag{3}$$

The probability of each answer $p(d|q)$ is determined by the weighted average probability of the answer over all hypotheses, $p(d|q) = \sum_{h \in H} p(d|h;q)p(h)$. For example, in Fig. 2, the probability of the answer "one" is $\frac{1}{4}$, the probability of the answer "two" is $\frac{1}{2}$, and the probability of the answer "three" is $\frac{1}{4}$. Thus, the EIG of the question "How many monsters have a square head?", given the hint in Fig. 2, is $(\frac{1}{4} * 2) + (\frac{1}{2} * 1) + (\frac{1}{4} * 2) = 1.5$.

Excluded Questions

We could only analyze questions that could be translated into the task-specific language, as the majority of our analyses operated directly on questions' program representations. As a result, we had to exclude responses that were invalid, ambiguous, or otherwise not able to be represented in the task-specific language.

Invalid responses were those that did not follow the instructions for the task. This includes non-questions, questions that could require a multi-word answer, off-topic questions, and questions that queried irrelevant features of the monsters. For example: "Can you tell me how are all of the monsters?", "What do the purple, blue, and red monsters do?", "Three legs.", "What is your favorite animal?".

Ambiguous responses were questions that did not have a single clear meaning. For example, the question "What's the shape of its head?" is ambiguous in a trial where multiple monsters' heads are covered. This category also included questions like "Does the red monster look like the blue monster?" (ambiguous whether "looks like" means the same head, legs, or either/both), and "Does the purple or blue monster have an even number of

legs?" (if the purple monster has an even number of legs but the blue monster does not, it is unclear whether the answer would be "yes" or "the purple monster").

Finally, a very small number of questions could not be represented in the task-specific language. This included questions like "What monster, if any, is the only one with their unique head shape?", "How many shape sides and legs total are there between the three monsters?", and "Assigning a value of 10 to square and 20 to circle, and keeping the number of legs equal to that many points, what is the total number of points between all three monsters?".

Exclusion rates were higher among children than for adults. In Study 1, 11% of children asked no valid questions across the four trials, while 0% of adults asked no valid questions. We fit a mixed-effects logistic regression model predicting response exclusion on a trial-by-trial basis, with a fixed effect for age group (children, adults) and by-participant and by-trial random intercepts. This revealed a significant difference in odds of trial exclusion between children and adults, $\chi^2(1) = 4.26$, $p = .04$, $OR = 0.36$, 95% CI $[0.13, 0.97]$.

We found similar results in Study 2. Again, 11% of children asked no valid questions across the four trials, while only 2% of adults asked no valid questions. In addition, there was a significant effect of age group (5- to 6-year-olds, 7- to 8-year-olds, 9- to 10-year-olds, adults) on the odds of trial-level exclusion, $\chi^2(3) = 44.29$, $p < .001$. Regression coefficients are reported in Table 3.

Table 3

Study 2, odds ratios (with 95% CIs) for effect of age group (reference group: 5- to 6-year-olds) on the odds of trial exclusion.

	Dependent variable:
	Excluded response
Age Group [7- to 8-year-olds]	0.15^{**} (0.05, 0.48)
Age Group [9- to 10-year-olds]	0.13^{***} (0.04, 0.43)
Age Group [Adults]	0.04^{***} (0.01, 0.12)
Intercept	0.46 (0.20, 1.05)
Note:	*p<0.05; **p<0.01; ***p<0.001

Validating Task-Specific Language Translations

As described above, all questions were translated into the task-specific language. To guard against errors in the translation process, we validated these translations by back translating all question-programs into natural language questions using GPT-3.5-turbo (Brown et al., 2020). A trained research assistant then compared the original question against the back translated question. Inconsistencies were resolved by the first author (and through discussion with the other authors as needed).

The prompt provided to GPT-3.5-turbo was as follows:

I'm translating questions into computer programs. Below, I'm including some examples.

[EXAMPLES]

Here is another program; can you tell me the corresponding question?

Program: *[QUESTION PROGRAM]*
Question:

For each study, the prompt included a set of example questions and translations. We used different examples for each study and age group (children, adults) by randomly selecting 15 questions from within the relevant dataset. These 15 example translations were independently checked by a trained research assistant to ensure accuracy.

For Study 2, we conducted the translation validation process twice: once upon initial translation of the questions into programs, and again after changing how a particular type of question was translated. Specifically, the questions "How many monsters have [shape] head?" / "How many monsters have [number] legs?" were initially translated using the `setSize` function (e.g., `(setSize (shapeObjects Square))`. However, we later noticed that similar question templates (e.g., "Do any monsters have [shape] head?") were instead translated using lambda expressions (e.g., `(any (map (lambda x0 (== (shape x0) Square)) (set Blue Red Purple)))`). We decided to consistently use the latter format for all questions of this form. To ensure we made no errors in the re-translation process, we conducted a second round of GPT-assisted validation.

This validation procedure identified very few errors in the original translations. For Study 1, all conflicts between the original and back translated questions were resolved in favor of the original translations. For Study 2 (summing across both rounds of validation), two translation errors were corrected, and two questions were reclassified as ambiguous.

Can Participants Understand the Target Questions in Study 2?

One possible concern about Study 2's procedure is that participants may not have understood the target questions: "How many monsters have a square head?" and "How many legs do all the monsters have combined together?" If the target question (and its answer) are not comprehensible to a participant, we could not reasonably expect that participant to reuse and recombine the target question. To address this concern, we tested whether participants (in the exposure condition) answered the confederate's question correctly when she first posed the target question. If participants did not answer the target question correctly, this suggests they may have had difficulty understanding it.

We found that most participants answered the target question correctly. First, we fit a logistic regression model predicting whether a child participant answered the target question correctly, as a function of age (in months), target question condition, and their interaction. There was no evidence for an interaction between age and target question condition, $\chi^2(1) = 1.83$, $p = .18$. In a model excluding the interaction term, there was no evidence for an effect of target question condition (reference group: "How many monsters

have a square head?"), $\chi^2(1) = 0.46$, $p = 0.50$, $OR = 1.65$, 95% CI $[0.39, 7.67]$. However, there was a significant effect of age (in months) within childhood, $\chi^2(1) = 11.13$, $p < .001$, $OR = 1.07$, 95% CI $[1.03, 1.13]$: with increasing age, participants were more likely to provide a correct answer to the target question. The target question was answered correctly by 82% of 5- to 6-year-olds, 94% of 7- to 8-year-olds, and 100% of 9- to 10-year-olds. Notably, participants were presented with four answer choices when responding to the confederate's question: thus chance responding would result in 25% accuracy. Thus, even the youngest age group were far above chance accuracy, $\chi^2(1) = 56.82$, $p < .001$.

Though there was evidence for an effect of age within childhood, 9- to 10-year-olds' uniform success made it impossible to obtain reliable regression results in a model using binned age group (5- to 6-year-olds, 7- to 8-year-olds, 9- to 10-year-olds, adults). Therefore, we next fit a logistic regression model predicting correct answers, with age group (children, adults), target question condition, and their interaction as predictors. Again, there was no evidence for a significant interaction, $\chi^2(1) = 0.27$, $p = .60$, so we dropped the interaction term from the model. In the absence of the interaction term, there was no evidence for an effect of either age group, $\chi^2(1) = 0.37$, $p = .54$, $OR = 1.39$, 95% CI $[0.48, 4.26]$, or target question condition, $\chi^2(1) = 0.87$, $p = .35$, $OR = 1.65$, 95% CI $[0.58, 5.07]$. 95% of adults answered the target question correctly.

In summary, participants were able to correctly answer (and thus seemed to comprehend) the target question. While children's success at answering the target question increased between 5 and 10 years of age, even the youngest participants had far higher than chance accuracy. Moreover, accuracy did not differ by target question condition, suggesting that the two questions were similarly comprehensible.

Alternative Analysis of Reuse

In Study 2, our main analyses of reuse used logistic regression models, predicting whether or not the asked question on each trial matched the target question. However, in some age groups and conditions, there were cases where no participants asked target-matching questions at all. To verify the robustness of our results, we also conducted an additional analysis where we model the *number* of target-matching questions asked (summed across the four trials for each participant).

We fit the following model, with age referring to binned age group (5- to 6-year-olds, 7- to 8-year-olds, 9- to 10-year-olds, adults):

*number of target-matching questions ~ (exposure condition + previous quality condition + target question) * age + (1 | participant id)*

First, we found no evidence for a significant interaction between exposure condition and age group, $\chi^2(3) = 7.05$, $p = 0.07$. However, when excluding the interaction term from the model, there was a main effect of exposure condition, $\chi^2(1) = 47.85$, $p < .001$, $b = 0.47$, 95% CI $[0.33, 0.60]$. Participants asked more target-matching questions in the exposure condition compared to the no-exposure condition. Thus, like our main analyses, there was evidence for a reuse effect that did not vary in magnitude across development.

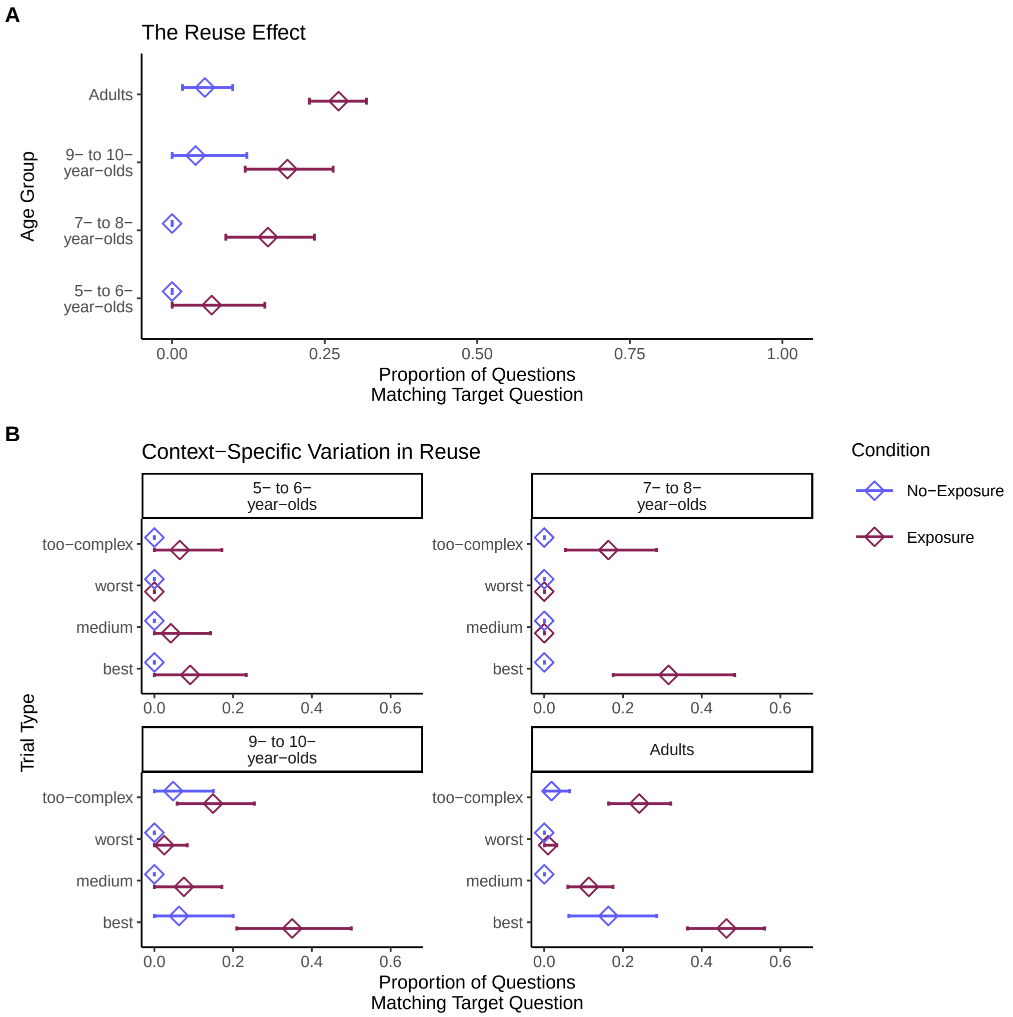

Figure 3

***A:** Frequency of target-matching questions (with bootstrap 95% CIs), in the no-exposure versus exposure condition in each age group (only for the best, medium, and too-complex trial types, where the target question had informational value). **B:** Frequency of target-matching questions (with bootstrap 95% CIs), across the four trial types for each age group. Y-axis truncated for ease of visualization.*

Additional Figures

To help contextualize the results of Study 2, we plot the reuse effect and the recombination effect across binned age group (breaking up children's data into three bins: 5- to 6-year-olds, 7- to 8-year-olds, 9- to 10-year-olds). This is shown in Fig. 3 and Fig. 4. Note that the effects of age within childhood were not statistically significant at the preregistered alpha = .01 level.

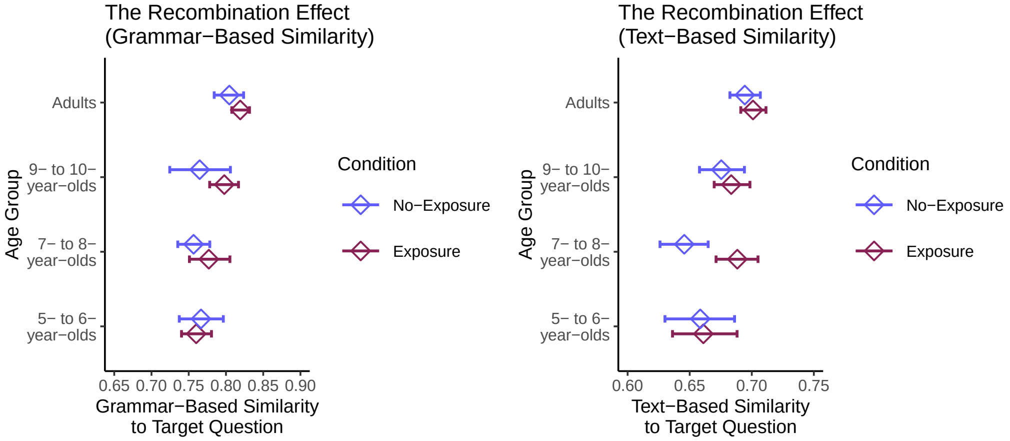

Figure 4

Mean similarity (with bootstrap 95% CIs) between asked questions and target question, including only questions that did not match the target question, in the no-exposure versus exposure condition for each age group. Left panel uses grammar-based similarity; right panel uses text-based similarity. Y-axis truncated for ease of visualization.

Why do People Reuse Less When Questions are Less Informative?

One open question from this work is the mechanism underlying context-specific reuse. We found that children and adults were less likely to reuse the target question when that question was less informative in the current situation. Is this because the target question fails to come to mind when it is not informative? Or is this because the target question comes to mind but is discarded due to its low informational value?

One way to test these possibilities is to investigate the degree of recombination across situations. If the target question fails to come to mind when it is not informative, we would expect lower amounts of recombination on these trials, as well. That is because there is no target question in mind to recombine. In contrast, if the target question comes to mind equally across trials but then is discarded when it is not informative, we would expect similar amounts of recombination across trials.

To test these possibilities, we asked whether the recombination effect (the difference in target-question similarity between the exposure condition and the no-exposure condition) varied across trial types. We fit the following linear mixed-effects model:

> *target-question similarity* $\sim$ *exposure condition* * *trial type* + *(exposure condition* + *previous quality condition* + *trial type* + *target question)* * *age group* + *(1 | participant id)*

We tested whether there was evidence for an interaction between exposure condition and trial type, controlling for all other manipulations and their interactions with age group (children, adults). For grammar-based similarity, there was no evidence for an interaction between exposure condition and trial type, $\chi^2(3) = 7.27$, $p = .06$. Likewise, for

text-based similarity, there was no evidence for an interaction between exposure condition and trial type, $\chi^2(3) = 5.98$, $p = .11$. Though the interactions were not significant, we also computed estimated marginal means for each model to estimate the effect of exposure condition (no-exposure vs. exposure) within each trial type. For both measures of recombination, the estimated condition contrast was largest in the worst trial (grammar-based similarity: 0.04, 95% CI $[0.01, 0.06]$; text-based similarity: 0.03, 95% CI $[0.01, 0.05]$). In comparison, the estimated condition contrast was smallest in the too-complex trial for grammar-based similarity (-0.001 95% CI $[-0.03, 0.02]$) and the best trial for text-based similarity (-0.003, 95% CI $[-0.03, 0.02]$). This is opposite what would be expected if the target question failed to come to mind when it was not informative (e.g., in the worst trial). Rather than observing less recombination on those trials, we observed the largest recombination effects on those trials. This is consistent with the possibility that the target question came to mind across all trials, but was discarded in favor of innovation when it was not informative.

Study 1: Full regression results

In Study 1, we reported regression coefficients for models with only dichotomous categorical predictors. Here, in Tables 4 and 5, we report the full set of coefficients for models that include categorical predictors with multiple levels.

Table 4

Study 1, regression coefficients (with 95% CIs) for effect of age group (reference group: 5- to 6-year-olds) on the number of unique functions used in a question's program representation.

	Dependent variable:
	Number of unique functions
Age Group [7- to 8-year-olds]	0.70* (0.01, 1.40)
Age Group [9- to 10-year-olds]	1.34*** (0.62, 2.05)
Age Group [Adults]	1.25*** (0.56, 1.94)
Intercept	2.11*** (1.50, 2.72)
Note:	*p<0.05; **p<0.01; ***p<0.001

Table 5

Study 1, regression coefficients (with 95% CIs) for effect of age group (reference group: 5- to 6-year-olds) on grammar-based similarity between an asked question and the most-similar previous question asked by the same participant (i.e., across-trial recombination).

	Dependent variable:
	Grammar-based similarity to most-similar previous question
Age Group [7- to 8-year-olds]	−0.10 (−0.21, 0.01)
Age Group [9- to 10-year-olds]	−0.15* (−0.26, −0.03)
Age Group [Adults]	−0.16** (−0.26, −0.05)
Intercept	0.85*** (0.76, 0.93)
Note:	*p<0.05; **p<0.01; ***p<0.001

Study 2: Full regression results

In Study 2, we only reported regression coefficients for specific effects of interest. Here, we report the full regression results. These results are from the final models reported in the manuscript, where we dropped non-significant interaction terms for the effects of interest (i.e., "focal effects"). Interaction terms were retained for any non-focal effect. All of the following regression models use contrast coding for categorical variables (e.g., for categorical variables with two levels, we code one level as -0.5 and the other level as 0.5). This allows for easy interpretation of all regression coefficients even in the presence of interaction terms. For each categorical variable, the intercept represents the average value of the outcome variable averaged across levels of the categorical variable. Each additional contrast compares one level of the categorical variable (indicated in brackets) to a reference group (the unmentioned level). In all cases, the target question “Legs" refers to the question “How many legs do all the monsters have combined together?”.

Tables 6 and 7 report analyses of reuse. Tables 8, 9, 10, and 11 report analyses of recombination. We did not have specific predictions about how trial type would affect recombination. However, the results shown here demonstrate that recombination did vary across trial types—differentially for grammar-based similarity compared to text-based similarity. As measured by grammar-based similarity (Table 10), questions asked in the too-complex trial were less similar to the target question, compared to questions asked in the best trial. Moreover, questions asked in the medium trial were slightly more similar to the target question, compared to questions asked in the best trial. These patterns were reversed when measured by text-based similarity (Table 11): questions asked in the too-complex trial were more similar to the target question, compared to questions asked in the best trial. Questions asked in the medium trial and the worst trial were less similar to the

target question, compared to questions asked in the best trial.

Table 12 reports the regression coefficients for a model testing the effect of exposure condition on question EIG. Finally, Tables 13 and 14 report analyses of across-trial reuse and recombination.

Table 6

Study 2, odds ratios (with 95% CIs) predicting whether an asked question matches the target question. Focal effect: Exposure Condition.

	Dependent variable:
	Match to target question
Exposure Condition [Exposure]	10.75^{***} (4.92, 23.49)
Previous Quality Condition [Previously Informative]	0.84 (0.52, 1.36)
Age Group [Adults]	2.93^{***} (1.65, 5.22)
Trial Type [Medium]	0.09^{***} (0.04, 0.18)
Trial Type [Too-Complex]	0.31^{***} (0.19, 0.50)
Question Condition [Legs]	1.28 (0.79, 2.07)
Previous Quality Condition [Good] : Age Group [Adults]	1.43 (0.54, 3.79)
Age Group [Adults] : Trial Type [Medium]	1.05 (0.27, 3.99)
Age Group [Adults] : Trial Type [Too-Complex]	0.70 (0.28, 1.77)
Age Group [Adults] : Question Condition [Legs]	1.02 (0.39, 2.67)
Intercept	0.05^{***} (0.03, 0.08)
Note:	$^{*}p<0.05$; $^{**}p<0.01$; $^{***}p<0.001$

Table 7

Study 2, odds ratios (with 95% CIs) predicting whether an asked question matches the target question (in the exposure condition only). Focal effects: Previous Quality Condition and Trial Type.

	Dependent variable:
	Match to target question
Previous Quality Condition [Previously Informative]	0.87 (0.55, 1.37)
Trial Type [Medium]	0.11*** (0.06, 0.21)
Trial Type [Too-Complex]	0.34*** (0.21, 0.55)
Trial Type [Worst]	0.01*** (0.003, 0.05)
Question Condition [Legs]	1.70* (1.06, 2.71)
Age Group [Adults]	2.41*** (1.48, 3.90)
Question Condition [Legs] : Age Group [Adults]	1.21 (0.47, 3.08)
Intercept	0.08*** (0.05, 0.13)
Note:	*p<0.05; **p<0.01; ***p<0.001

Table 8

Study 2, regression coefficients (with 95% CIs) predicting grammar-based similarity between an asked question and the target question (excluding questions that use the same template as the target question). Focal Effect: Exposure Condition.

	Dependent variable:
	Grammar-based similarity to target question
Exposure Condition [Exposure]	0.02* (0.003, 0.03)
Previous Quality Condition [Previously Informative]	−0.01 (−0.03, 0.002)
Age Group [Adults]	0.04*** (0.03, 0.06)
Trial Type [Medium]	0.01 (−0.003, 0.03)
Trial Type [Too-Complex]	−0.06*** (−0.08, −0.05)
Trial Type [Worst]	−0.01 (−0.02, 0.01)
Question Condition [Legs]	0.02*** (0.01, 0.04)
Previous Quality Condition [Previously Informative] : Age Group [Adults]	0.01 (−0.02, 0.04)
Age Group [Adults] : Trial Type [Medium]	0.01 (−0.02, 0.04)
Age Group [Adults] : Trial Type [Too-Complex]	−0.06*** (−0.09, −0.03)
Age Group [Adults] : Trial Type [Worst]	0.01 (−0.02, 0.04)
Age Group [Adults] : Question Condition [Legs]	−0.004 (−0.03, 0.02)
Intercept	0.79*** (0.79, 0.80)
Note:	*p<0.05; **p<0.01; ***p<0.001

Table 9

Study 2, regression coefficients (with 95% CIs) predicting text-based similarity between an asked question and the target question (excluding questions that use the same template as the target question). Focal Effect: Exposure Condition.

	Dependent variable:
	Text-based similarity
Exposure Condition [Exposure]	0.02^{**} (0.005, 0.03)
Previous Quality Condition [Previously Informative]	0.001 (−0.01, 0.01)
Age Group [Adults]	0.03^{***} (0.02, 0.04)
Trial Type [Medium]	-0.05^{***} (−0.06, −0.03)
Trial Type [Too-Complex]	0.03^{***} (0.02, 0.04)
Trial Type [Worst]	-0.13^{***} (−0.14, −0.11)
Question Condition [Legs]	-0.01^{**} (−0.03, −0.004)
Previous Quality Condition [Previously Informative] : Age Group [Adults]	0.002 (−0.02, 0.02)
Age Group [Adults] : Trial Type [Medium]	0.02 (−0.01, 0.05)
Age Group [Adults] : Trial Type [Too-Complex]	−0.003 (−0.03, 0.03)
Age Group [Adults] : Trial Type [Worst]	0.03^{*} (0.004, 0.06)
Age Group [Adults] : Question Condition [Legs]	0.003 (−0.02, 0.03)
Intercept	0.69^{***} (0.68, 0.69)
Note:	$^{*}p<0.05$; $^{**}p<0.01$; $^{***}p<0.001$

Table 10

Study 2, regression coefficients (with 95% CIs) predicting grammar-based similarity between an asked question and the target question (in the exposure condition only; excluding questions that use the same template as the target question). Focal Effect: Previous Quality Condition.

	Dependent variable:
	Grammar-based similarity
Previous Quality Condition [Previously Informative]	−0.02 (−0.03, 0.001)
Trial Type [Medium]	0.02^{*} (0.001, 0.04)
Trial Type [Too-Complex]	-0.06^{***} (−0.09, −0.04)
Trial Type [Worst]	0.01 (−0.02, 0.03)
Age Group [Adults]	0.04^{***} (0.02, 0.05)
Question Condition [Legs]	0.01 (−0.01, 0.03)
Trial Type [Medium]: Age Group [Adults]	0.03 (−0.01, 0.08)
Trial Type [Too-Complex] : Age Group [Adults]	−0.04 (−0.08, 0.01)
Trial Type [Worst] : Age Group [Adults]	0.04 (−0.01, 0.08)
Age Group [Adults] : Question Condition [Legs]	−0.004 (−0.04, 0.03)
Intercept	0.80^{***} (0.79, 0.81)
Note:	$^{*}p<0.05$; $^{**}p<0.01$; $^{***}p<0.001$

Table 11

Study 2, regression coefficients (with 95% CIs) predicting text-based similarity between an asked question and the target question (in the exposure condition only; excluding questions that use the same template as the target question). Focal Effect: Previous Quality Condition.

	Dependent variable:
	Text-based similarity
Previous Quality Condition [Previously Informative]	0.002 (−0.01, 0.02)
Trial Type [Medium]	−0.04*** (−0.06, −0.02)
Trial Type [Too-Complex]	0.03*** (0.01, 0.05)
Trial Type [Worst]	−0.11*** (−0.13, −0.10)
Age Group [Adults]	0.02** (0.01, 0.04)
Question Condition [Legs]	−0.03*** (−0.04, −0.01)
Trial Type [Medium]: Age Group [Adults]	0.02 (−0.02, 0.06)
Trial Type [Too-Complex] : Age Group [Adults]	−0.01 (−0.04, 0.03)
Trial Type [Worst] : Age Group [Adults]	0.04* (0.002, 0.07)
Age Group [Adults] : Question Condition [Legs]	−0.01 (−0.04, 0.02)
Intercept	0.69*** (0.69, 0.70)
Note:	*p<0.05; **p<0.01; ***p<0.001

Table 12

Study 2, regression coefficients (with 95% CIs) predicting question informativeness (EIG). Focal Effects: Exposure Condition and Age.

	Dependent variable:
	EIG
Exposure Condition [Exposure]	0.10** (0.03, 0.17)
Previous Quality Condition [Previously Informative]	0.02 (−0.04, 0.09)
Age Group [Adults]	0.30*** (0.24, 0.37)
Trial Type [Medium]	−0.09** (−0.16, −0.03)
Trial Type [Too-Complex]	−0.21*** (−0.27, −0.14)
Trial Type [Worst]	−0.25*** (−0.31, −0.18)
Question Condition [Legs]	0.13*** (0.06, 0.20)
Previous Quality Condition [Previously Informative] : Age Group [Adults]	0.03 (−0.10, 0.16)
Age Group [Adults] : Trial Type [Medium]	0.01 (−0.12, 0.14)
Age Group [Adults] : Trial Type [Too-Complex]	−0.23*** (−0.36, −0.10)
Age Group [Adults] : Trial Type [Worst]	−0.15* (−0.28, −0.02)
Age Group [Adults] : Question Condition [Legs]	0.05 (−0.09, 0.18)
Intercept	1.23*** (1.19, 1.26)
Note:	*p<0.05; **p<0.01; ***p<0.001

Table 13

Study 2, regression coefficients (with 95% CIs) for effect of age group (reference group: 5- to 6-year-olds) on across-trial reuse.

	Dependent variable:
	Match to any previously-asked question
Age Group [7- to 8-year-olds]	0.66 (0.40, 1.09)
Age Group [9- to 10-year-olds]	0.42** (0.25, 0.71)
Age Group [Adults]	0.52** (0.34, 0.80)
Intercept	0.81 (0.55, 1.19)
Note:	*p<0.05; **p<0.01; ***p<0.001

Table 14

Study 2, regression coefficients (with 95% CIs) for effect of age group (reference group: 5- to 6-year-olds) on grammar-based similarity between an asked question and the most-similar previous question asked by the same participant (i.e., across-trial recombination).

	Dependent variable:
	Grammar-based similarity to most-similar previous question
Age Group [7- to 8-year-olds]	−0.02 (−0.06, 0.02)
Age Group [9- to 10-year-olds]	−0.06** (−0.09, −0.02)
Age Group [Adults]	−0.05** (−0.09, −0.02)
Intercept	0.88*** (0.85, 0.91)
Note:	*p<0.05; **p<0.01; ***p<0.001

References

Brown, T. B., Mann, B., Ryder, N., Subbiah, M., Kaplan, J., Dhariwal, P., Neelakantan, A., Shyam, P., Sastry, G., Askell, A., Agarwal, S., Herbert-Voss, A., Krueger, G., Henighan, T., Child, R., Ramesh, A., Ziegler, D. M., Wu, J., Winter, C., … Amodei, D. (2020). Language Models are Few-Shot Learners [arXiv]. https://doi.org/10.48550/arXiv.2005.14165

Lindley, D. V. (1956). On a measure of the information provided by an experiment. *The Annals of Mathematical Statistics*, *27*(4), 986–1005. https://doi.org/10.1214/aoms/1177728069

Liquin, E. G., & Gureckis, T. M. (2022). Where questions come from: Reusing old questions in new situations. In J. Culbertson, A. Perfors, H. Rabagliati, & V. Ramenzoni (Eds.), *Proceedings of the 44th Annual Conference of the Cognitive Science Society* (pp. 1160–1167). Cognitive Science Society.

Oaksford, M., & Chater, N. (1994). A rational analysis of the selection task as optimal data selection. *Psychological Review*, *101*(4), 608–631. https://doi.org/10.1037/0033-295X.101.4.608

Reimers, N., & Gurevych, I. (2019). Sentence-BERT: Sentence embeddings using siamese BERT-networks [arXiv]. https://doi.org/https://doi.org/10.48550/arXiv.1908.10084

Rothe, A., Lake, B. M., & Gureckis, T. (2017). Question asking as program generation. *Advances in Neural Information Processing Systems 30*, 1046–1055.

Rothe, A., Lake, B. M., & Gureckis, T. M. (2018). Do people ask good questions? *Computational Brain & Behavior*, *1*(1), 69–89. https://doi.org/10.1007/s42113-018-0005-5

Ruggeri, A., Lombrozo, T., Griffiths, T. L., & Xu, F. (2016). Sources of developmental change in the efficiency of information search. *Developmental Psychology*, *52*(12), 2159–2173. https://doi.org/10.1037/dev0000240

Shannon, C. E. (1948). A mathematical theory of communication. *The Bell System Technical Journal*, *27*(3), 379–423. https://doi.org/10.1002/j.1538-7305.1948.tb01338.x

Zhang, K., & Shasha, D. (1989). Simple Fast Algorithms for the Editing Distance between Trees and Related Problems. *SIAM Journal on Computing*, *18*(6), 1245–1262. https://doi.org/10.1137/0218082
